# Enhanced Sestrin expression through Tanshinone 2A treatment improves PI3K-dependent inhibition of glioma growth

**DOI:** 10.1038/s41420-023-01462-6

**Published:** 2023-05-19

**Authors:** Judith Schaf, Sonia Shinhmar, Qingyu Zeng, Olivier E. Pardo, Philip Beesley, Nelofer Syed, Robin S. B. Williams

**Affiliations:** 1 Centre for Biomedical Sciences, School of Biological Sciences, Royal Holloway University of London, Egham, TW20 0EX UK; 2 John Fulcher Neuro-Oncology Laboratory, Imperial College London, Hammersmith Hospital, London, UK; 3 Division of Cancer, Department of Surgery and Cancer, Imperial College London, London, UK

**Keywords:** Cell signalling, Cancer models, Drug development

## Abstract

Glioblastomas are a highly aggressive cancer type which respond poorly to current pharmaceutical treatments, thus novel therapeutic approaches need to be investigated. One such approach involves the use of the bioactive natural product Tanshinone IIA (T2A) derived from the Chinese herb Danshen, where mechanistic insight for this anti-cancer agent is needed to validate its use. Here, we employ a tractable model system, *Dictyostelium discoideum*, to provide this insight. T2A potently inhibits cellular proliferation of *Dictyostelium*, suggesting molecular targets in this model. We show that T2A rapidly reduces phosphoinositide 3 kinase (PI3K) and protein kinase B (PKB) activity, but surprisingly, the downstream complex mechanistic target of rapamycin complex 1 (mTORC1) is only inhibited following chronic treatment. Investigating regulators of mTORC1, including PKB, tuberous sclerosis complex (TSC), and AMP-activated protein kinase (AMPK), suggests these enzymes were not responsible for this effect, implicating an additional molecular mechanism of T2A. We identify this mechanism as the increased expression of *sestrin*, a negative regulator of mTORC1. We further show that combinatory treatment using a PI3K inhibitor and T2A gives rise to a synergistic inhibition of cell proliferation. We then translate our findings to human and mouse-derived glioblastoma cell lines, where both a PI3K inhibitor (Paxalisib) and T2A reduces glioblastoma proliferation in monolayer cultures and in spheroid expansion, with combinatory treatment significantly enhancing this effect. Thus, we propose a new approach for cancer treatment, including glioblastomas, through combinatory treatment with PI3K inhibitors and T2A.

## Introduction

Tanshinones are a group of bioactive components found in the herbal extract Danshen, derived from the root of *Salvia miltiorrhiza Bunge* that has been used for centuries in traditional Chinese medicine [1]. The most abundant of these lipophilic compounds is Tanshinone 2A (T2A) [2], that reduces tumour cell growth in cancer models [3–5] and tumour growth in vivo [6]. Although numerous underlying molecular mechanisms have been reported for T2A as an anti-cancer agent, multiple studies have proposed a therapeutic mechanism through the inhibition of the phosphoinositide 3 kinase (PI3K) and protein kinase B (PKB), to inhibit the mechanistic target of rapamycin complex 1 (mTORC1) signalling pathway [5, 7–9]. Alternatively, it has also been proposed that T2A may function by increasing the activity of mTORC1 negative regulators including AMP-activated protein kinase (AMPK) [4], Tuberous Sclerosis Complex 2 (TSC2) and Sestrin [4]. Thus, improved insight regarding these mechanism(s) may help in the development of new approaches for the treatment of cancers.

Glioblastoma multiforme (GBM) is an aggressive, grade 4 cancer that affects 3.21 in 100,000 people [10], representing 15% of brain tumour diagnoses [11]. Patients diagnosed with GBM have a 5% survival chance of 5 years, increasing the demand for more effective and novel therapies [12]. T2A has been used in investigations to treat GBM development, where it inhibits proliferation and stemness [3, 13, 14] or decreased neuropathic pain observed as a side-effect of platinum based chemotherapy [15]. Several mechanisms have been proposed for how T2A inhibits GBM growth, beyond PI3K/PKB/mTORC1 signalling, including through the inhibition of STAT3 and increased activation of death receptors [13, 16] or through upregulation of neural lineage marker expression [3], but further mechanistic insight may provide new approaches for GBM treatment.

The use of a tractable model systems with reduced genetic redundancy and the ability to genetically ablate these pathways has been very valuable in providing improved insight into the molecular mechanism(s) of therapeutic treatments [17–20]. As one such model, *Dictyostelium discoideum*, contains various proteins and signalling pathways that are evolutionary conserved in humans and that have been linked to various diseases, such as cancer and related treatments [21]. Using this model, a range of phenotypes including cell proliferation inhibition or signalling pathway regulation can be monitored to assess the activity of drugs and medicine natural products, while the ablation of target genes can help characterise their molecular mechanisms. In particular, *D. discoideum* has been employed as a model system to analyse the effects of therapeutic compounds on mTORC1 activity, to identify the associated underlying molecular mechanisms and validate these mechanisms in patient-derived cells [19, 20] and in resulting clinical tials [22, 23].

In this study, we analysed the molecular and cellular mechanisms of T2A. Initially employing *D. discoideum*, we demonstrate that T2A treatment reduces cell proliferation, and acutely blocks PI3K and PKB activity. Surprisingly however, only chronic T2A treatment inhibits the activity of mTORC1. This chronic effect was independent of commonly associated mTORC1 regulators, PKB, TSC2 and AMPK, but involved the increase of sestrin (*sesn*) expression. Indeed, loss of *sesn* prevented the T2A-dependent inhibition of cell proliferation and mTORC1 activity. Furthermore, combinatory treatment with T2A and a PI3K inhibitor provided synergistic inhibition of cell proliferation. Translation of this combination to human and mouse glioblastoma 2D cell and spheroid cultures reproduced these effects on cell proliferation, with the efficacy of a clinical PI3K inhibitor (Paxalisib) being significantly enhanced by addition of T2A.

## Results

### T2A reduces *D. discoideum* cell proliferation

**Fig. 1:**
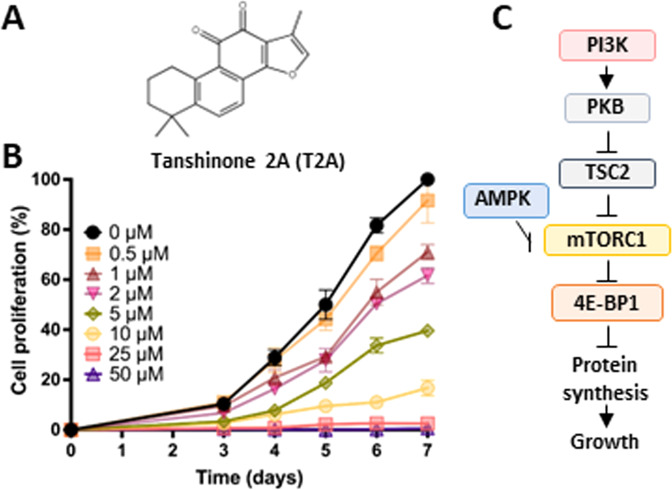
T2A reduces *D. discoideum* cell proliferation and has been implicated in regulating the conserved PI3K/PKB/mTORC1 signalling pathway. **A** Tanshinone 2A (T2A) is a diterpene quinone extracted from the medicinal herb Danshen. **B** Cellular proliferation of wild type *D. discoideum* cells was reduced at increasing concentrations of T2A over a seven-day period. *N* = 6 (3 independent, 2 technical repeats). All data are shown as mean ± SEM; **p ≤ 0.01, ***p ≤ 0.001 (two-tailed Mann–Whitney test- unpaired and non-parametric data). **C** The PI3K/PKB/mTORC1 signalling pathway controls cell proliferation, has been implicated as a target for T2A, and is conserved in *D. discoideum*, suggesting that this model can be used to investigate the molecular effects of T2A.

To validate *D. discoideum* as a suitable model to investigate the cellular activity and molecular mechanisms of T2A (Fig. 1A), we first analysed the dose-dependent effects of this compound on cellular proliferation. Wild type cells show a dose-dependent reduction in cell proliferation (Fig. 1B), with near-maximal inhibition (96.0%) at 16 µM (p < 0.001), and an IC_50_ of 2.7 µM, consistent with concentrations used in mammalian studies [4, 8, 24]. Since T2A has been suggested to act through the PI3K/PKB/mTORC1 signalling pathway (Fig. 1C), that is largely evolutionarily conserved between *D. discoideum* and humans [25, 26], changes to the activity of this pathway in our model was next analysed in response to T2A.

### T2A acutely inhibits PI3K activity

**Fig. 2:**
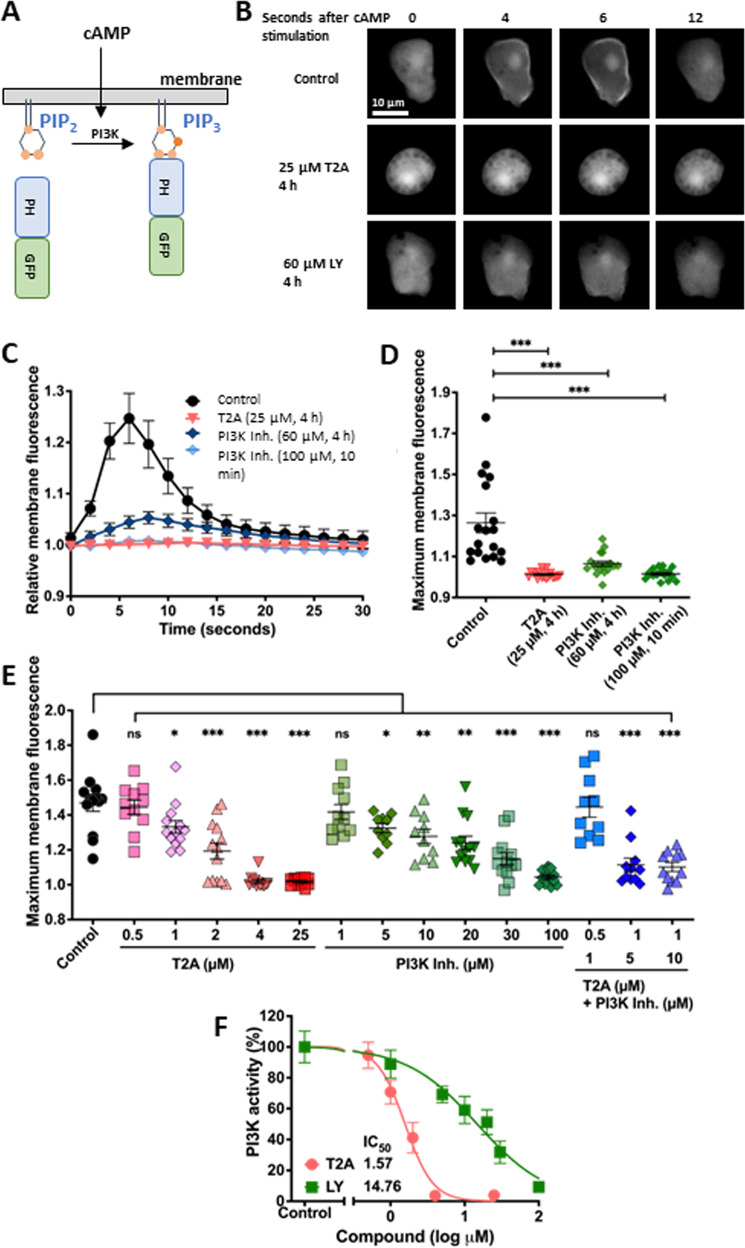
T2A acutely inhibits PI3K activity in *D. discoideum*. **A** PI3K activity can be quantified in *D. discoideum* by assessing the translocation of the PH_Crac_-GFP to bind PIP_3_ on the membrane following stimulation with single pulse of cAMP (6 biological repeats, 3 technical repeats). **B** Under control (solvent only) conditions PIP_3_-induced membrane localisation of PH_Crac_-GFP peaks at 4–6 s after cAMP stimulation. This activity is lost following treatment with T2A (25 µM for 4 h) or a PI3K inhibitor (LY294002; 60 µM for 4 h) (Supplementary Fig. S2) (6 biological repeats, 3 technical repeats). **C** Quantification of PIP_3_ production over time, in the presence of T2A or LY294002, by transient PH_Crac_-GFP membrane localisation, and **D** Maximum membrane fluorescence value confirms T2A inhibition of PI3K activity. **E** Dose-dependent inhibition of PIP_3_ production by T2A (0–25 µM) and LY294002 (0–100 µM) with 15 min treatment indicated by maximum membrane fluorescence values, identified a dose dependent acute block in PIP_3_ production (Supplementary Fig. S3) (6 biological repeats, 3 technical repeats). The combinations of 1 µM T2A with 5 µM PI3K inhibitor and 1 µM T2A with 10 µM PI3K inhibitor resulted in synergistic inhibition of PI3K activity (Supplementary fig. 3 for BLISS analysis) (#). **F** Comparison of PI3K inhibition by T2A and LY294002, enabling calculation of relative potency (IC_50_ values). All data are shown are derived from at least *N* = 6, with three independently stimulated cells analysed per experiment, and presented as mean ± SEM; *p ≤ 0.05, **p ≤ 0.01, ***p ≤ 0.001, ns (not significant, *p* > 0.05) (two-tailed Mann–Whitney test), # shows synergy (BLISS analysis).

To analyse the effects of T2A on the PI3K/PKB/mTORC1 signalling pathway in *D. discoideum*, we initially focused on the first step in this pathway, PI3K. In *D. discoideum*, a single pulse of cAMP (10 μM) functions to activate PI3K, generating transient phosphatidylinositol (3,4,5)-trisphosphate (PIP_3_) at the plasma membrane, that can be quantified using the temporal translocation of an expressed PH_Crac_-GFP protein from the cytosol to the cell membrane [27] (Fig. 2A). We found that PIP_3_ is rapidly and transiently produced in response to the pulse, with translocation of PH_Crac_-GFP to the membrane within ~5 second and returns to the cytosol ~12 seconds later (Fig. 2B). Both T2A (25 µM for 4 h), and the PI3K inhibitor LY294002 (at 60 μM for 4 h or 100 μM for 10 min) blocked PIP_3_ production (Fig. 2C, D, Supplementary Fig. S1). These inhibitory effects were dose dependent following acute treatment (15 min), with T2A providing greater potency than LY294002 (Fig. 2E, F, Supplementary Fig. S2). The combination treatments of 1 μM T2A with 5 μM LY294002 and 1 μM T2A with 10 μM LY294002 synergistically inhibited the activity of PI3K, as defined by BLISS analysis (supplementary Fig. 3) [28]. T2A treatment reduced PI3K activity with an IC_50_ value of 1.57 µM (Fig. 2F), providing a 96.3% (p < 0.001) reduction at 4 µM. In contrast, the IC_50_ for LY294002 was 14.8 µM (Fig. 2F), with a 90.8% reduction (p < 0.001) obtained at 100 µM, suggesting that T2A is more potent at inhibiting PI3K. In addition, combined treatment with T2A (1 µM) and LY294002 (5 µM or 10 µM), provides a synergistic reduction in PI3K activity. These experiments demonstrate that the PI3K-inhibitory effect of T2A, which has previoulsy been shown in cancer studies [5, 8] is conserved in *D. discoideum*.

### T2A acutely inhibits PKB activity

**Fig. 3:**
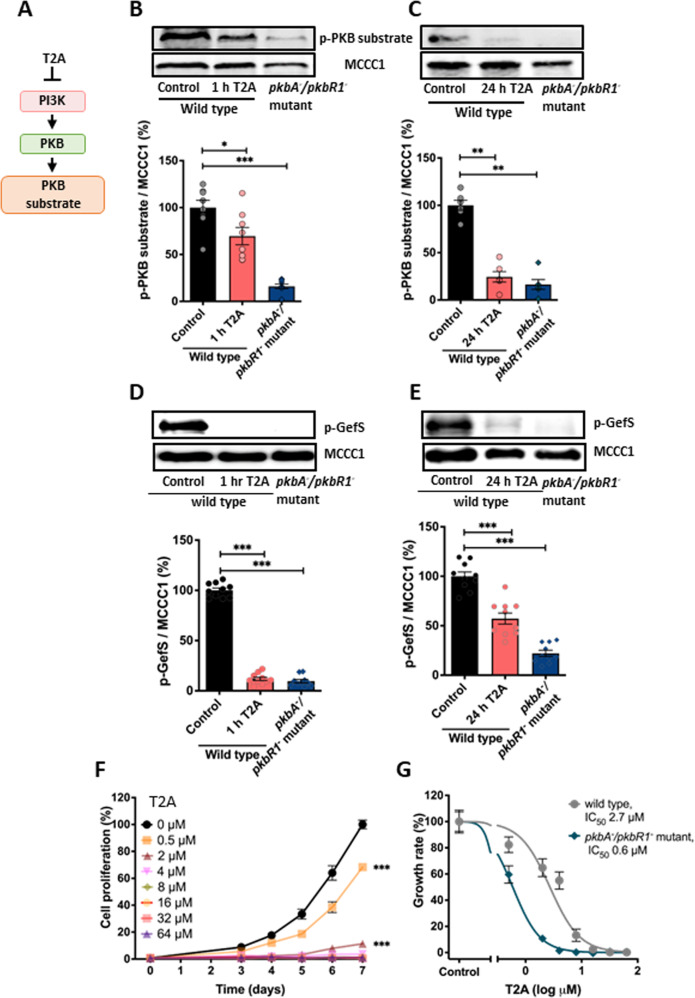
T2A acutely reduces PKB activity, but loss of PKB sensitises cell to T2A proliferation inhibition. **A** T2A-dependent PI3K inhibition would be expected to inhibit PKB activity, measured by phosphorylation of specific substrates. **B**, **C** Analysis of PKB activity by monitoring substrate phosphorylation, following treatment with T2A (25 μM) for **B** 1 h or **C** 24 h relative to the loading control MCCC1, *n* ≥ 6 (b. 3 biological repeats, 2 technical for 2, and one technical for another. c. 4 biological repeats). **D**, **E** Analysis of p-GefS phosphorylation following treatment with T2A (25 μM) for **D** 1 h or **E** 24 h relative to loading control MCCC1, *n* ≥ 6. **F** Analysis of T2A-sensitive cell proliferation in cells lacking PKB activity (*pkbA*^*-*^*/pkbR1*^*-*^), *N* ≥ 4 (at least 4 biological repeats with 2 technical repeats each). **G** Effect of T2A on growth rate enables comparison of potency (IC_50_) for wild type and the *pkbA*^*-*^*/pkbR1*^*-*^ mutant (4 biological repeats, 2 technical repeats each). All data are shown as mean ± SEM; *p ≤ 0.05, **p ≤ 0.01, ***p ≤ 0.001, ns (not significant, *p* > 0.05) (two-tailed Mann–Whitney test).

Since PI3K activity leads to the direct downstream activation of the serine/threonine-specific protein kinase B (PKB), and T2A reduces PKB activation in a variety of mammalian models [4, 7, 8, 29–31], we then assessed the effect of T2A on PKB activity in *D. discoideum* (Fig. 3A). Here cells were treated for 1 or 24 h using a concentration of T2A that blocks PI3K activity (25 µM), and PKB activity was assessed by measuring PKB output through monitoring the phosphorylation level of its substrates using a consensus sequence-directed antibody (p-PKB substrate) [32] and of the guanine nucleotide exchange factor S (GefS) [33]. T2A treatment reduced general phosphorylation of PKB substrates, reducing it by 30.4% (p < 0.05) (Fig. 3B) and 75.8% (p < 0.01) (Fig. 3C) following 1 or 24 h, respectively, with similar effects seen with GefS phosphorylation levels, where acute treatment reduced protein levels to 11% and chronic treatment to 57% (Fig. 3D-E).

Since the effect of T2A on PI3K/PKB signalling may underlie the observed inhibition of cell proliferation, we then assessed the dependency of this phenotype on the presence of PKB. Here, we took advantage of the low redundancy genome of *D. discoideum*, containing two PKB-encoding genes, and employed a double *pkbA*^*-*^*/pkbR1*^*-*^ null mutant to analyse how PKB modulates the activity of T2A (Fig. 3F, G). Surprisingly, in these cells, treatment with T2A at 4 µM produced a 96.4% reduction in cell proliferation (p < 0.001) (Fig. 3F), and a half maximal inhibitory concentration (IC_50_) of 0.6 µM (Fig. 3G), indicating increased sensitivity to T2A as compared to that observed in wild type cells. These results suggest that T2A is likely to act through at least two cellular mechanisms; one involving PI3K/PKB inhibition and a second independent mechanism relating to cell proliferation. Investigating this second mechanism may provide new understanding of the molecular effects of T2A.

### Chronic treatment with T2A inhibits mTORC1 activity, independently of PKB and AMPK signalling

**Fig. 4:**
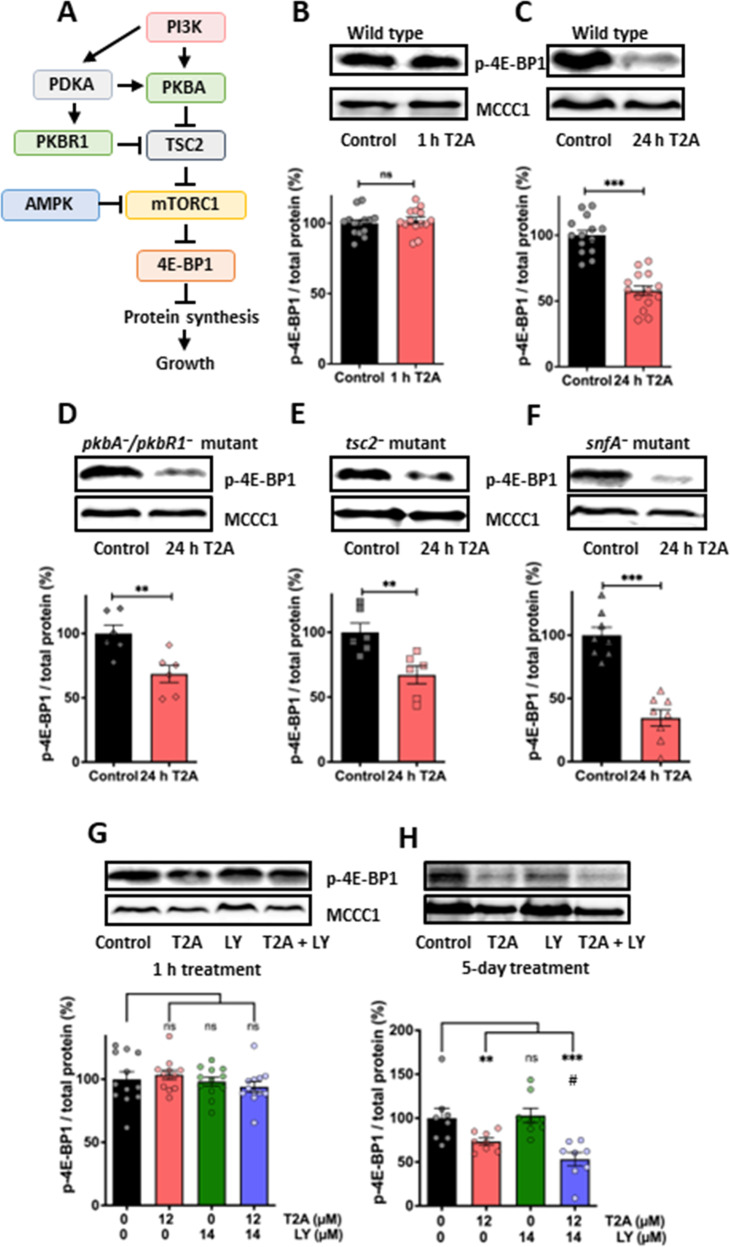
Chronic T2A treatment inhibits mTORC1 activity independent of PKB, TSC2 and AMPKα. **A** Multiple pathways of mTORC1 regulation have been proposed, including through PI3K and PKB (in *D. discoideum*, PKBA and PKBR1) and AMPK signalling, suggesting one of these pathways may control the T2A-dependent reduction of mTORC1 activity. In these experiments mTORC1 activity was measured by the quantification of the phosphorylated substrate (4E-BP1). T2A (25 μM) treatment of wild type cells for **B** 1 h does not inhibit mTORC1 activity, but **C** 24 h treatment significantly reduced this activity. This T2A-dependent mTORC1 inhibition was independent of **D** PKB activity (in the *pkbA*^*-*^*/pkbR1*^-^ mutant), **E** TSC2 activity (in the *tsc2*^-^ mutant) and **F** AMPKα (in the *snfA*^*-*^ mutant) where T2A (25 µM) significantly reduced 4E-BP1 phosphorylation. **G** Inhibition of PI3K activity (14 μM LY294002) does not reduce mTORC1 activity in 1 h, nor in combination with T2A (12 μM), however, **H** extended treatment (5 days) indicates that T2A (12 μM) reduces mTORC1 activity and provides enhanced inhibitory effects in combination with a PI3K inhibitor (14 μM LY294002) significantly reduced compared to T2A only treatment (#). Data is shown using MCCC1 as a loading control. *N* ≥ 6. All data are shown as mean ± SEM; */#p ≤ 0.05, **p ≤ 0.01, ***p ≤ 0.001, ns (not significant, *p* > 0.05) (two-tailed Mann–Whitney test).

**Fig. 5:**
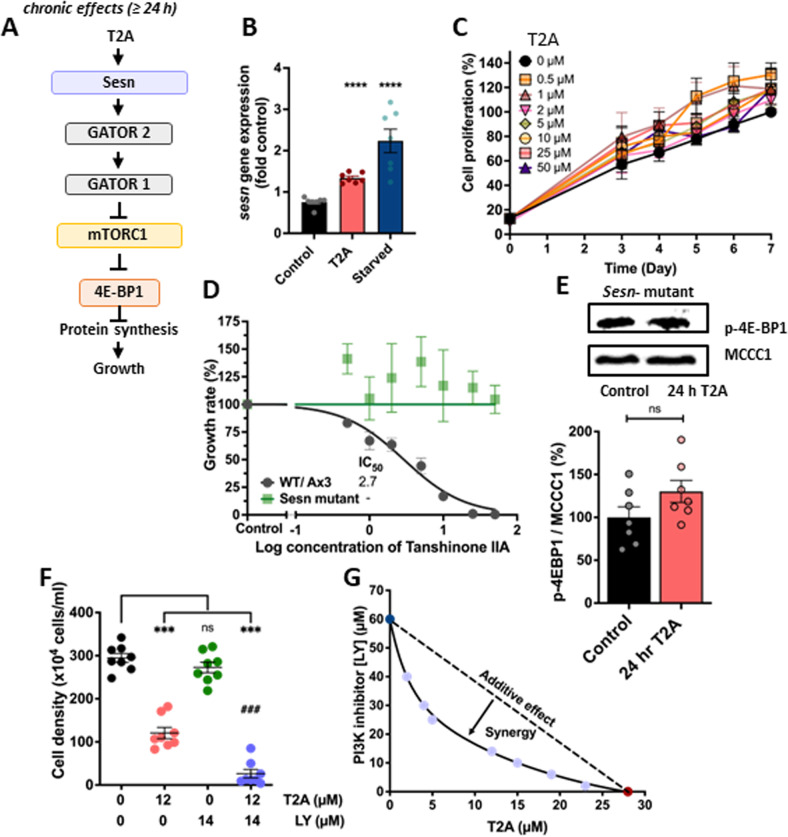
Chronic T2A treatment enhances sestrin expression to reduce cell proliferation. **A** Sestrin (*sesn*) functions through GATOR2 and 1 complex to inhibit mTORC1 activity. **B** To analyse potential changes in Sestrin expression, wild type cells were exposed to T2A (3 days; 25 μM) or starvation (4 h), and gene expression was assessed by qPCR, where both T2A treatment and starvation increased expression by 33 and 223% respectively (3 biological repeats, at least 2 technical repeats each). **C**, **D** To assess the effect of loss of Sestrin on T2A-dependent proliferation reduction, wild type and *sesn*^*-*^ mutant cell growth was assessed (days 3–7) where the mutant was unaffected at saturating T2A concentrations (50 μM) (3 independent repeats, 3 technical repeats). **E** Analysis of T2A-dependent mTORC1 inhibition in *sesn*^*-*^ mutant cells exposed to control (solvent only) conditions or following 24 h T2A (25 µM) treatment no reduction of p-4E-BP1 levels using MCCC1 as a loading control, indicating a loss of T2A-dependent mTORC1 inhibition in the mutant (3 biological repeats, at least 2 technical repeats each). **F** Analysis of cell proliferation effects of T2A (12 µM), the PI3K inhibitors LY296004 (14 µM) and combination of both over 5 days, where combinatory treatment provided (4 biological repeats, 2 technical repeats each), **G** synergistic inhibition of cell proliferation following 5-day treatment, shown using isobolographic analysis at a 90% reduction of cell proliferation. Data is derived from *N* = 6 (**B**–**D**) *n* = 7 (**E**–**G**). All data are shown as mean ± SEM, ns = *p* > 0.05, *p ≤ 0.05, **p ≤ 0.01, ***p ≤ 0.005 (two-tailed Mann–Whitney test).

Since PI3K/PKB activity regulates the mTORC1 complex [26] (Fig. 4A), which is a key pharmacological target in cancer treatment, and we show here that PI3K/PKB activity is inhibited by T2A treatment [8, 30], we investigated the effect of T2A on mTORC1 in *D. discoideum*. mTORC1 activity was assessed by Western blotting analysis of phosphorylation of one of its downstream substrates 4E-BP1 [19, 20], as mTORC1-specific antibodies are not available for *D. discoideum* samples, following 1 or 24 h treatment with T2A (25 µM) (Fig. 4B, C). In these assays, 1 h treatment did not reduce mTORC1 activity (Fig. 4B), but 24 h treatment caused a 42.1% reduction (Fig. 4C). To investigate this reduction, we again employed a range of null mutants lacking upstream regulators of mTORC1 and treated these cells for 24 h with T2A (25 µM). Here, T2A treatment continued to reduce mTORC1 activity in the absence of PKB activity (31.5% reduction in *pkbA*^*-*^*/pkbR1*^*-*^ cells; Fig. 4D), TSC activity [34] (32.9%, Fig. 4E) and 5’-AMP-activated serine/threonine-protein kinase (AMPK) activity (65.5% reduction in *snfA*^*-*^ cells [19], Fig. 4F). Furthermore, neither LY294002, T2A nor a combination of both agents reduced mTORC1 activity following 1 h treatment (Fig. 4G), confirming the absence of acute inhibition through this pathway. Since genetic ablation of PKB enhanced T2A-mediated inhibition of cell proliferation over 7 days, we also investigated the effect of 5 days treatment with LY294002, T2A (12 µM) and their combination on mTORC1 activity. This showed that while LY294002 (14 µM) had no effect, an unexpectedly strong inhibition of mTORC1 activity was obtained with the combinatory treatment (Fig. 4H) which provided synergistic inhibition of mTORC1 signalling [28]. Taken together, these results suggest that acute treatment with T2A reduces PI3K/PKB signalling without immediate effects on mTORC1 activity. However, long term T2A treatment provides mTORC1 inhibition independently of PI3K/PKB and AMPK signalling, and combination treatment of T2A with a PI3K inhibitor further enhance this effect.

### T2A increases *sesn* expression and, in combination with PI3K inhibition, synergistically reduces cell proliferation

Sestrin (*sesn*) expression increases in starvation or following cellular stress, to inhibit mTORC1 through GATOR1/2 complexes [35] (Fig. 5A). This pathway has been demonstrated in *D. discoideum*, where increased expression of the single *sesn* orthologue (DDB_G0279427) has been shown to reduce mTORC1 activity [36]. To investigate a role for T2A in controlling this pathway, *sesn* expression was assessed following T2A treatment (25 µM, 24 h). This showed that *sesn* expression was increased by 33% following T2A treatment (Fig. 5B). This suggests that T2A may reduce cell proliferation through inducing *sesn* expression. To test this hypothesis, the Sestrin gene was ablated (Supplementary Fig. S4) and compared to wild type cells for their response to T2A. These experiments revealed the resulting *sesn*^-^ mutant to have a strongly reduced proliferation rate as compared to wild type cells (Supplementary Fig. S4). Treatment with T2A (up to 50 μM) did not lead to additional loss of cell proliferation (Fig. 5C, D), consistent with the fact that mTORC1 activity in this mutant was resistant to T2A treatment (Fig. 5E). These results suggest that increased *sesn* expression downstream of T2A treatment is responsible for the downregulation of mTORC1 activity and associated reduction in cell proliferation.

Since our data suggest the potential effectiveness of combining T2A and LY294002 treatment (Fig. 4H), we investigated the effectiveness of this combination on cell proliferation of wild type *D. discoideum* as compared to single treatments. These experiments show that while LY294002 treatment (14 µM) had no effect on cell proliferation, its combination with T2A (12 µM) further decreased cell proliferation as compared to T2A treatment alone in a way that appeared to be above additive (Fig. 5F). To assess the potential for synergistic interaction between the two compounds, an isobolographic analysis was performed using a range of T2A and LY294002 to provide a 90% reduction in cell proliferation (Fig. 5G). This confirmed a strong synergistic effect of the two compounds in reducing cell proliferation in *D. discoideum*.

### Combinatory T2A and PI3K inhibitor treatment inhibits GBM cell proliferation

**Fig. 6:**
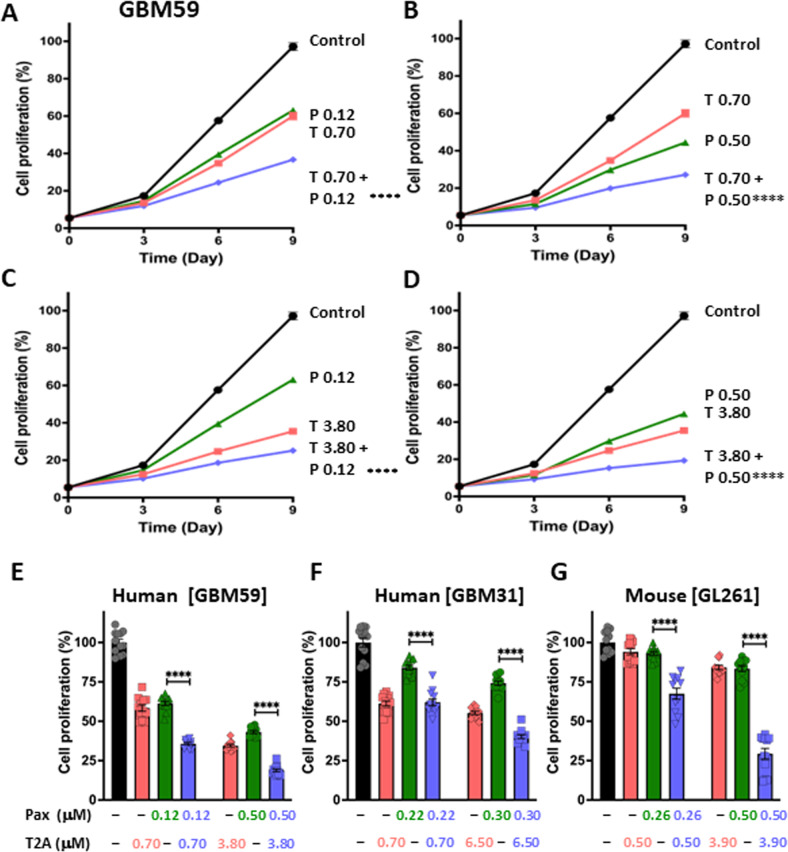
T2A enhances Paxalisib-dependent inhibition of human- and mouse-derived GBM cell proliferation. Human-derived GMB cells (GBM59) were cultured in a range of T2A (2.5–40 μM) and Paxalisib (0.25–2 μM) concentrations and cell proliferation was measured over 9 days. **A**–**E** GBM59 cells were exposed to a low and high concentration of each compound as well as combinatory treatments, where cell proliferation inhibition was significantly enhanced compared to Paxalisib alone. **A**–**D** shows kinetic representations of the cell proliferation over 9 days of treatment. The same effect of enhanced cell proliferation inhibition can be seen for **F** GBM31 and **G** GL261 cells, where a combinatory treatment of both high and low concentration of Paxalisib and T2A resulted in greater inhibition of cell proliferation compared to Paxalisib alone. Data is derived from 3 biological repeats, with each experiment comprising 12 technical repeats. All data are shown as mean ± SEM, ns = *p* > 0.05, ****p ≤ 0.0001 (Mann–Whitney test).

To translate our discoveries from *D. discoideum* to relevant pre-clinical models, we investigated combinatory T2A and PI3K inhibitors in the reduction of Glioblastoma multiforme (GBM) cell proliferation. GBM is an aggressive cancer derived from astrocytes [10], with poor clinical outcomes [37], and one new approach for treating GBM patients involves the use of the PI3K inhibitor Paxalisib, a potent, oral, selective, brain penetrant class 1 phosphoinositide 3-kinase inhibitor [38]. We therefore initially investigated whether combination treatment with Paxalisib and T2A would reduce GBM cellular proliferation in monolayer cell cultures. Here, we employed two patient-derived GBM cell lines (GBM59 and GBM31 [39]) and one mouse cell line (GL261 [40]) exposed to a range of T2A and Paxalisib concentrations. We tested the effect of combining T2A and Paxalisib, using varied concentrations of each component to reflect the potential variation in clinical use. In the human GBM59 cell line (Fig. 6A-D) cell proliferation was measured from day 0 to day 9 in cells treated with T2A (0.70–3.80 μM) and Paxalisib (0.12–0.50 μM) and combinations (T 0.70 + P 0.12, T 0.70 + P 0.50, T 3.80 + P 0.12 and T 3.80 + P 0.50). In each treatment group, combination significantly reduced cell proliferation compared to Paxalisib monotreatment. Analysis of proliferation on day 9 was measured where in the GBM59 cell line, Paxalisib treatment at 0.12 μM and 0.5 μM reduced cell proliferation to 61 and 43% of control respectively (Fig. 6E), and T2A at 0.7 μM and 3.8 μM reduced cell proliferation to 58 and 34% respectively, consistent with earlier studies [3, 6]. Combinatory treatment using both high and low doses respectively enhanced the effect of Paxalisib by around two-fold, providing a reduction in cell proliferation to 36 and 19% in each case (Fig. 6E). Similarly, Paxalisib treatment of the human GBM31 cell line at 0.22 and 0.3 μM provided a reduction in cell proliferation to 84 and 74% of control respectively, where this reduction was enhanced with the addition of 0.7 or 6.5 μM T2A to 62 and 40% (Fig. 6F, Supplementary Fig. S5). Finally, treatment of the mouse GL261 cell line with Paxalisib at 0.26 or 0.5 μM provided a reduction in cell proliferation to 93 and 83% of control respectively, while this reduction was enhanced with the addition of 0.5 or 3.9 μM T2A resulting in 68 and 29% of the proliferation of control cells respectively (Fig. 6G, Supplementary Fig. S5). In both human- and mouse-derived glioblastoma cell lines, combinatory treatment with T2A and Paxalisib provided significantly enhanced reduction of glioblastoma proliferation in comparison to Paxalisib alone treatment.

### Combinatory T2A and PI3K inhibitor treatment inhibits GBM spheroids expansion

**Fig. 7:**
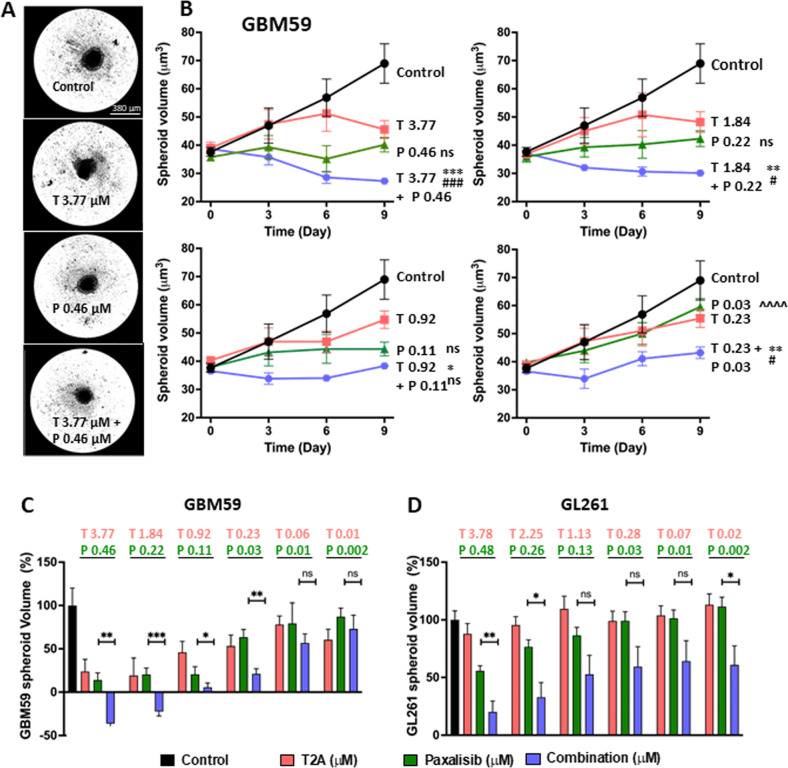
T2A enhances Paxalisib-dependent inhibition of human- and mouse-derived GBM spheroid expansion. **A** Human-derived glioblastoma cells (GBM59) were cultured into 3D spheroids and grown under control conditions (solvent only), in the presence of T2A (3.77 µM), Paxalisib (0.46 µM) and in combination, where each treatment reduced spheroid volume and combined treatment provided an enhanced reduction. **B** Quantification of GBM59 spheroid volumes, following exposed to a range of T2A (T: 3.77–0.01 µM) and Paxalisib (P: 0.46–0.002 µM) concentrations and in combinations, where each combinatory treatment provided significantly improve spheroid reduction effects to Paxalisib alone (*p < 0.05, **p < 0.01, **p < 0.001). The two higher dose combinations provided a significant reduction in volume compared to the initial volume (#*p* < 0.05, ###*P* < 0.001) (see also Supplementary Fig. S7). **C**, **D** Comparison of normalised spheroid volume following 9day treatment grown under control conditions (solvent only), in the presence of T2A or Paxalisib, and in combination, where **C** GBM59 and **D** mouse-derived GL261 spheroids showed significant volume reduction following by the addition of T2A to Paxalisib in a range of concentrations. All data are shown as mean ± SEM (3 biological repeats, 3 technical repeats each), with statistical analysis using two-tailed Mann–Whitney test.

Since tumour-based cancer growth is better reflected using 3D models to reproduce cellular microenvironment, spatial organisation and cell-cell communication within tumours, we investigated the effects of Paxalisib and T2A individually and in combinations on spheroids [41] derived from GBM59 (human) and GL261 (mouse) cells [42] excluding GBM31 cells due to inability to form spheroid cultures. Single spheroids were prepared in individual wells (Fig. 7A) and exposed to a range of treatments. Spheroid volume was estimated prior to and at day 3, 6 and 9 of treatment. Following 9 days treatment at multiple concentrations of both Paxalisib (0.11–0.46 μM) and T2A (0.92–3.77μM), spheroid volumes were heavily reduced compared to control conditions (Fig. 7A, B, Supplementary Fig. S6), while lower concentrations (0.03 μM, and 0.23 μM respectively) had limited effects. A total of 6 different combinations of T2A and Paxalisib were tested on GBM59 spheroids (Fig. 7C), with the 4 highest concentration combinations significantly decreasing growth above that of Paxalisib alone treatment. In addition, the two highest combinations lead to spheroid growth regression, a phenotype not observed with either compound used alone. Consistent with these results, significant increase in growth reduction was observed for combinations of Paxalisib and T2A over single agent treatment in GL261 spheroids (Fig. 7D, Supplementary Fig. S7). BLISS values were calculated to determine potential synergy of combinatory treatments on the inhibition of spheroid volume growth [28]. The combinations of Tanshinone IIA and Paxalisib together enhanced the growth inhibitory effect of treatment in a synergistic capacity (Supplementary Fig S8). In this 3D cancer model, the human-derived glioblastoma spheroids showed greater sensitivity to T2A and combined treatment than the mouse-derived model, although both showed improved reduction in spheroid volume with combinatory treatment.

### T2A treatment increases sestrin2 gene expression and protein levels in GBM cell lines

**Fig. 8:**
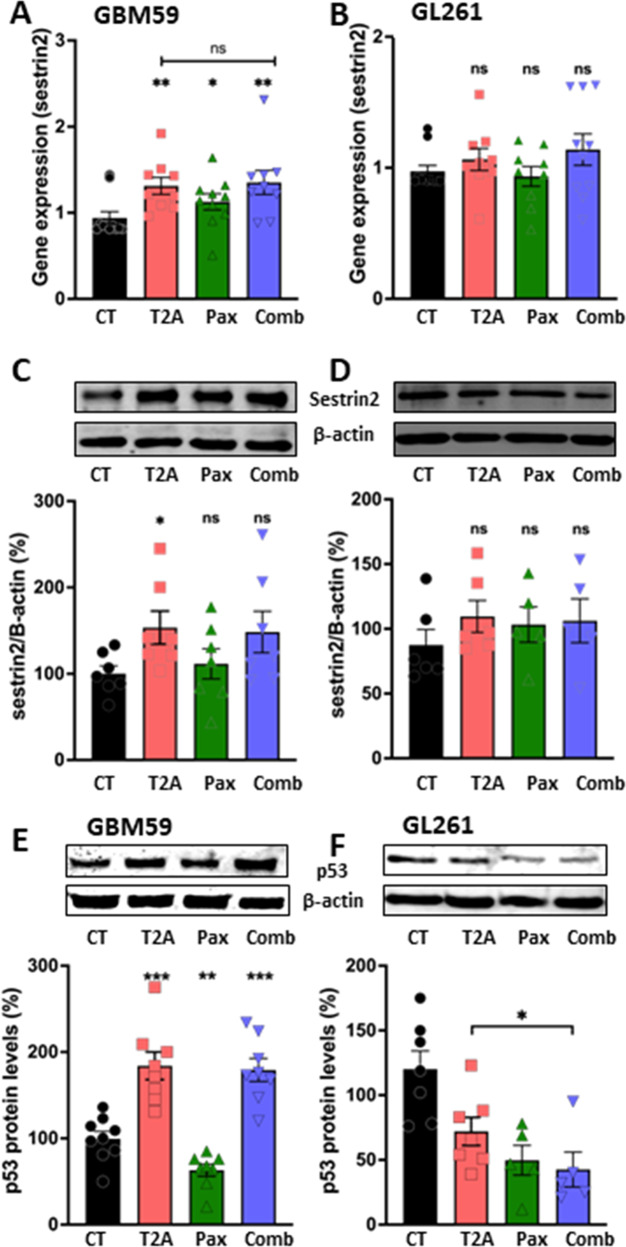
T2A treatment enhances Sestrin2 expression in GBM59 cells. GBM59 and GL261 cells were grown in 2D cell culture conditions and treated with T2A (3.77 μM), Paxalisib (0.46 μM) or in combination for 3 days and resulting Sestrin gene expression was measured by qPCR and Sestrin2 protein levels by western analysis. **A** In GBM59 cells, Sestrin expression increased by 31% following T2A treatment, by 13% following Paxalisib treatment, and by 51% following combination treatment. **B** In GL261 cells, following treatment with T2A (3.78 μM), Paxalisib (0.48 μM) or in combination for 3 days, and no significant increase in sestrin2 expression levels were identified. **C** In GBM59 cells, under similar conditions, Sestrin2 protein levels increased by 53% following T2A treatment, while Paxalisib and combination treatments did not alter Sestrin2 protein levels. **D** In GL261 cells, under similar conditions, T2A, Paxalisib, or the combinatory treatment for did not significantly alter protein levels. β-actin was used as a loading control. **E** GBM59 cells, treated the same as before, showed increased p53 protein levels following T2A treatment and combination, however not with Paxalisib concentration alone. **F** GL261 cells did not increase p53 protein levels following treatment with Tanshinone IIA. β-actin was used as a loading control (3 biological repeats, 3 technical repeats for GBM59 and 2/3 technical repeats for GL261 each). Both E and F are normalised to loading control (β-actin). All data are shown as mean ± SEM; ns, not significant, *p* > 0.05, *p ≤ 0.05, **p ≤ 0.01, ***p ≤ 0.001, (two-tailed Mann–Whitney test).

Since both GBM59 and GL261 showed synergistic inhibition of growth with combined T2A and Paxalisib treatment, but all treatment provided greater growth inhibition in GBM59 cell, we investigated differential effect on Sestrin expression and protein levels, and p53 levels. In these experiments, both GBM59 and GL261 2D cultures were treated with T2A (3.77 μM), Paxalisib (0.46 μM) and in combination for 3 days, and gene expression was assessed by qPCR (Fig. 8A, B) and protein levels quantified by Western blot analysis (Fig. 8C, D). In GBM59 cells, Sestrin gene expression was increased by 33% with T2A treatment (P < 0.01), or by 50% in combination with Paxalisib (P < 0.01) but was not increased in GL261 cells under any condition. Similarly, in GBM59 cells, Sestrin2 protein levels increased by 53% with T2A treatment (P < 0.05) and did not increase in GL261 cells (Fig. 8C, D). Thus, T2A treatment provided a significant elevation in Sestrin levels in GBM59 cells, that was not found following treatment in GL261 cells. To investigate a mechanism for this difference, changes in p53 levels were analysed in both cell lines (Fig. 8E, F), where in GBM59 cells, p53 levels increased by 84% with T2A treatment (P < 0.01) and 79% by combinatory T2A and Paxalisib treatment (P < 0.01), but this increase was absent in GL261 cells. These data suggest combinatory T2A and Paxalisib treatment in responsive (GBM59) cells occurs through regulating p53 and Sestrin expression to provide extensive synergistic reduction in cell proliferation, with a reduced but still significant synergistic reduction in cell proliferation in less responsive (GL261) cells.

## Discussion

The onset and progression of many types of cancers are caused by the over-activation of the PI3K/PKB signalling pathway with the resultant activation of mTORC1 [43–45]. This is often caused by mutations within the pathway, that are, for example, responsible for 70% of colorectal and breast cancers [43, 46] and for ~80% of GBM cases [47], highlighting the importance of developing new treatments for cancer therapies that target this pathway. T2A has been shown to reduce the PI3K signalling pathway in numerous cancer-related studies [5, 7–9] leading to a reduction in downstream PKB and mTORC1 signalling [48]. In our study, we identify that in *D. discoideum* T2A treatment inhibits PI3K/PKB signalling, and mTORC1 activity. In defining this mechanism, we surprisingly show that inhibition of PI3K does not lead to a reduction in mTORC1 activity in this model, yet T2A treatment reduces mTORC1 activity independent of the known signalling pathway intermediates PKB, TSC and AMPK. We further show that T2A treatment enhances *sesn* expression, loss of the single *sesn* gene decreases the potency of T2A in reducing cell proliferation by at least 18-fold (with IC_50_ value in wild type cells increasing from 2.7 µM to >50 µM in the *sesn*^**-**^ mutant) and blocks the reduction of mTORC1 activity following T2A treatment. Thus, in this model, the mechanism of mTORC1 inhibition by T2A treatment unexpectedly appears to be through enhanced sestrin signalling that reduces mTORC1 activity and decreases cellular proliferation, independent of PI3K inhibition. Furthermore, combinatory treatment with T2A and PI3K inhibitors provides synergistic inhibition of cell proliferation. This discovery is consistent with human studies, where patients with acute myeloid leukaemia (AML) show reduced *sesn* expression and increased mTOR levels in peripheral blood and bone marrow [49], and T2A treatment reduces PI3K activity and increases *sesn* expression in related human osteosarcoma cells [4]. The synergistic effect of combinatory T2A and PI3K inhibitor treatment was then demonstrated in multiple models of GBM growth.

Inhibition of PI3K signalling provides a well-accepted approach for cancer treatment, although drugs working through this mechanisms may show a reduction in efficacy over extended treatment periods, and often encounter some difficulties in clinical studies [50]. Resistance to PI3K inhibitors can for instance arise through multiple mechanisms that reactivate the PI3K pathway [51]. Examples of this include the loss of Phosphatase and TENsin homologue (PTEN) expression to reduce the degradation of the PI3K product, PIP_3_ [52]. Alternatively, downstream inhibition of mTORC1 results in increased PKB activity through loss of a negative feedback loop, leading to increased expression of the Glucose transporter 1 (GLUT1) and 4 sugar transporters, increasing sugar uptake and facilitating cancer cell proliferation and survival [53]. Hence new approaches to target cancer cell proliferation beyond PI3K/PKB inhibition are needed, and combinatory therapy may provide this option whilst also reducing the likelihood of developing treatment resistance.

Combination therapy, involving two or more components which target different mechanisms of action, can result in a supra-additive effects [54]. Here, low doses of two or more drugs may provide target inhibition greater than that of a higher dose of a single molecule or reduce side effects associated with high-dose drugs [54, 55]. Our study demonstrates significantly enhanced effects of combination of PI3K inhibitors with T2A on reducing cell proliferation, with synergistic interaction seen in inhibition of proliferation of *D. discoideum*, and in human- and mouse-derived GBM cells in 2D and spheroid cultures. This is particularly important for GBM patients, where this brain cancer is generally drug-resistant with poor 5-year survival rate of 5% [41], and GBM growth shows sensitivity to PI3K inhibitor treatments, such as Paxalisib (GDC0084) [38], as well as to T2A treatment [3, 6]. We show that combinatory treatment in two human and one mouse-derived GBM cell line, either in 2D culture or grown as spheroids, provides a significantly enhanced inhibition of cell proliferation compared to Paxalisib only treatment. These data suggest a potentially beneficial effect of combining Paxalisib and T2A for the treatment of GBM growth in a clinical setting.

Research models of GBM often provide significant variability, likely reflecting variation in patient populations, and this may provide some insight into drug mechanisms. In our experiments, although combinatorial Paxalisib and T2A treatment provided synergistic inhibition of spheroid expansion in both the human and mouse-derived lines, compound potencies were greater in the human-derived GBM cells. This difference is likely to be linked to the significant increase in Sestrin levels in the human-derived GBM cells following treatment that is absent in the mouse line arising from a mutation in the oncogene PTEN [40]. One function of PTEN is to activate the transcription factor p53 to enhance sestrin expression [56, 57]. Therefore, one model for this effect is that T2A may function through a PTEN induced p53 activation to enhance Sestrin expression [56] and reduce cell proliferation. Thus, multiple mechanisms may underly the efficacy of combination treatment with T2A and PI3K inhibitors that may be analysed in further studies.

The potential clinical relevance of the current study is dependent on the validity of the concentrations of Paxalisib and T2A used. For Paxalisib, the first human phase I clinical trial in GBM patients employed treatment at 45 mg/day [57], and a single brain sample was collected from one patient by surgical removal, where the concentration of Paxalisib was 0.8 µM [58], supporting the clinical relevance of the concentrations of Paxalisib used in our studies. For T2A, many pre-clinical in vitro studies employ T2A levels of around 10 µM [59, 60]. T2A is brain penetrant [61] with brain concentrations 31% of that found in plasma [62], that can be further increased to 75% in the presence of P-glycoprotein (P-gp) drug efflux pump inhibitors. However, since T2A is poorly absorbed due to high lipophilicity, a water-soluble sulfonated version has been developed that is applied through injection [63–65] and has been extensively used in clinical practice for many years [66]. Blood levels of this compound, evidenced in a recent single *i.v*. dose clinical trial, show concentrations reaching 0.5–1.0 mgL^−1^ (up to 2.5 µM) [67]. In addition, oral bioavailability of T2A increases 8–10 fold through consumption of crude Tanshinones extract rather than purified T2A [16]. Based upon these results, concentrations used in this study for T2A are also likely to be relevant to clinical practice. As both compounds are currently used as treatments, it is therefore expected that our results could be rapidly translated into clinical practice.

Inactivation of mTORC1 through combinatory treatment with Paxalisib and T2A may provide a useful approach in treating other diseases arising from PI3K/PKB/mTORC1 dysregulation. These diseases include many cancer types such as blader [68], ovarian [69], breast [70, 71] and non-small cell lung cancers [72] as well as inflammatory diseases like restenosis and neointimal hyperplasia [73]. It may also provide novel approaches in the treatment of a variety of neurological diseases such as epilepsy [74–76], and autism spectrum disorder [77] and metabolic disorders including Type II Diabetes Mellitus [78]. Thus, using PI3K inhibitors and T2A in combination may be beneficial to the treatment of a range of diseases.

In summary, our data suggest that treatment with combinations of T2A and PI3K inhibitors inhibit the growth of patient-derived and murine glioblastoma cells, in 2D and 3D cultures. Clinical trial testing will be needed to evaluate the therapeutic usefulness of these combinations. Such combinations may also be considered for a range of other cancer types and mTOR-opathies to not only enhance therapeutic outcome but also reduce side effects through equivalent potency at reduced drug concentration.

## Materials and Methods

### Chemical compounds

T2A was provided by AdooQ Bioscience (A10890), LY294002 by Cambridge Bioscience (CAY70920), and GDC0084 / Paxalisib by Selleckchem (S8163).

### *D. discoideum* cell proliferation assays

Cell proliferation was measured in liquid culture (HL5 media, Formedium), maintained in the dark at 22 °C in 24-well plates (with 5000 cells in 500 μl HL5 media per well), and treated with varying concentrations of compounds at a constant solvent level (DMSO, 0.8%). Cells were incubated for seven days, and cell densities were determined from day 3 to day 7 and normalised to the solvent-only control.

### Quantification of cell signalling by Western blotting

*D. discoideum* cells were treated during proliferation for the indicated times with control (solvent only) or compound with all condition containing 0.2% solvent. Protein samples were prepared by directly lysing 7.5 × 10^7^ cells/ml cells in 2 x Laemmli buffer (0.004% bromophenol blue, 10% 2-mercaptoethanol, 20% glycerol, 4% SDS, 0.125 M Tris-HCl) followed by boiling at 96 °C for 6.5 min. GBM human and mouse cells were treated during proliferation as above where final cell concentration was 1 × 10^6^ cells/ml. Protein samples were prepared using RIPA buffer and protease cocktail inhibitor and stored in 6X loading buffer and boiled for 5 mins at 100 °C. 6 - 20 µl of each sample was separated by sodium dodecyl sulfate polyacrylamide gel electrophoresis (stacking gel: 5%, resolving gel: 10 - 15%). After proteins were transferred to the appropriate membrane (polyvinylidene difluoride or nitrocellulose, pore size: 0.2–0.4 µm; Thermo Fisher Scientific, Millipore, Amersham), membranes were stained with Ponceau S dye and then blocked for 1 h with 5% bovine serum albumin (in TBS) or intercept (TBS) blocking buffer (LI-COR). Membranes were incubated overnight at 4 °C with primary antibodies detecting phospho(Thr37/46)-4E-BP1 (1:1000, Cell Signalling Technology, 9459) [19], p-PKB substrate (1:1000, Cell Signalling Technology (10001), 23C8D2) [32], p-GefS substrate (1:1000, Cell Signalling Technology (9614), 110B7E) [33], sestrin (1:1000, Cell Signalling Technology (#10795) and p53 (1:1000, Cell Signalling Technology (9282 T). All primary antibodies were dissolved in 5% BSA (TBST) or intercept (TBS) blocking buffer containing 0.1% Tween 20. As a loading control, either streptavidin Alexa Fluor 680 conjugate (1:5000, Invitrogen, S21378) for methylcrotonyl-CoA carboxylase (MCCC1) [79], AlexaFlour 690 conjugate (1:5000, LICOR, 926–68070) for B-actin (Sigma, A228), or Ponceau S stained total protein was used. Membranes were washed with TBST and subsequently incubated for 1 h with IRDye800CW goat anti-rabbit IgG (1:10000, LI-COR) diluted in 5% BSA (in TBST) or in intercept (TBS) blocking buffer containing 0.1% Tween 20 and 0.01% SDS. After membranes were washed with TBST, protein levels were visualised using the Odyssey CLx imaging system (LI-COR). Levels of the protein of interest were either normalised to the MCCC1, β-actin or to total protein (Ponceau S stain).

### Quantification of PI3K activity

The activity of PI3K was assessed as described previously [27]. In brief, *D. discoideum* wild type (Ax3) cells expressing PH_Crac_-GFP were made chemotactically competent by repeated stimulation with cAMP (6 min pulses of 100 nM) for 4 hours and subsequent to this, the cellular location of PH_Crac_-GFP was visualised following a single pulse of 10 µM cAMP, using a fluorescence microscope recording images every 2 sec for 1 min. Compounds or control (solvent only, 0.2% DMSO) were either added during the 4 h pulsing step or 10 - 15 min prior to the time-lapse imaging. Fluorescent intensities on the cell membrane and in the cytosol were calculated as relative to the whole cell, and normalised to the fluorescence intensity before cAMP stimulation, fluorescence was quantified using ImageJ.

### Quantification of *sesn* expression

*D. discoideum* wild type (Ax3) cells were cultured to 3 × 10^5^ cells/ml and treated for 24 hours with 25 µM or 12 µM T2A, 14 µM LY249002, or a combination of 12 µM T2A and 14 µM LY249002 or with solvent only (DMSO,0.2%) as the control. GBM cells were cultured to 1 × 10^6^ cells/ml and treated for 3 days with 3.78–3.88 µM T2A, 0.48–0.46 µM Paxalisib or a combination or with solvent only (DMSO, 0.2%). Following treatment, RNA was extracted from the cells (Qiagen, 74104) and cDNA synthesised (Thermo Fisher Scientific, K1622 kit). *sesn* expression was analysed using qPCR and primers around 100 base pairs apart within the gene (Sigma- SYBR® Green Jumpstart™ Taq ReadyMix™), with levels compared to the housekeeping gene *mcfQ* (DDB_G0272346) or HPRT (Gene ID: 15452), with expression fold-change calculate using the ΔΔ-Ct method. For glioblastoma cell lines, cells were grown in 2D cell culture and treated for 24 hours with T2A, the RNA was extracted using the Qiagen (RNeasy 74104) kit and the cDNA was produced using Thermo Fisher Scientific (K1622) kit. (Supplementary methods).

### Generation *of sesn* null mutants in *D. discoideum*

Wild type (Ax3) cells were transformed with the pTM1285 plasmid encoding the *sesn* gene target sequences (Supplementary methods), designed to target exon 2 of the *sesn* gene, and knockout were generated as previously described [80]. Target gene sequences were analysed to identify mutations.

### GBM cell proliferation assays

GBM59 and GBM31 were primary cell lines derived from surgically removed fresh tumour tissues or stereotactic biopsies [39]. These tissues were initially minced through a strainer (BD Biosciences) resulting in a suspension of single cells which were rapidly treated with sterile water to remove red blood cells. Remaining single cells were cultured as primary cell lines and maintained in DMEM supplemented with F-12 nutrient mixture and 10% FBS. GL261, an established mouse cell line [40], was grown in Dulbecco’s Modified Eagle Medium (DMEM, Gibco) supplemented with 10% Foetal Bovine Serum (FBS, PAN-Biotech). Incubation refers to 37 °C with 5% CO_2_. All experiments for individual experiments were performed at the same time, providing direct comparison to specific treatments.

To examine the effect of compounds on glioblastoma cell proliferation in 2D cultures, GL261, GBM31 and GBM59 cell cultures were exposed to a range of T2A concentrations (2.5–40 µM) and Paxalisib (0.25–2 µM). Cell viability and proliferation were measured using the Sulforhodamine B assay (SRB, Sigma Aldrich). In these experiments, 500 cells per well were incubated in 100 µl growth media in 96 well plates. After 24 hours, individual or combinations thereof were added. Cells were incubated and proliferation was measured following TCA fixing and stained using 0.4% SRB in 1% acetic acid. Following drying, 10 mM Tris solution was used to dissolve the SRB dye and quantified at 490 nm (BioTek EL800 Microplate Reader).

Cultured spheroid expansion was used to examine the effect of compounds on glioblastoma cell proliferation in 3D growth. Spheroids were prepared using 5 × 10^7^ cells of each glioblastoma cell line, cultured in individual wells of a 96 well plate at 37 °C for 4 days to allow spheroid formation [42]. Spheroid diameter was measured using Echo Revolver microscope prior to compound treatment, then following Paxalisib, T2A or combinatory treatment on days 3, 6 and 9. Images were analysed using ImageJ to measure the area of each spheroid and calculate approximate spheroid size change based on a spherical volume.

### Statistics

The data is represented as mean ± SEM. Statistical significance between two groups was analysed using a two-tailed Mann–Whitney test (GraphPad PRISM) as the data is mostly unpaired and non-parametric. N number selection is based on previously published studies from our group.

## Supplementary information

Supplementary information

Original Data File
