# Supplementary material for: Enhanced Sestrin expression through Tanshinone 2A treatment improves PI3K-dependent inhibition of glioma growth
Source: Cell Death Discov. 2023 May 19;9:172. doi: 10.1038/s41420-023-01462-6 (PMC10195868; doi:10.1038/s41420-023-01462-6)
Supplement: Supplementary file 1 — Supplementary information [file 41420_2023_1462_MOESM1_ESM.docx]

**Enhanced Sestrin expression through Tanshinone 2A treatment improves PI3K-dependent inhibition of glioma growth**

*Judith Schaf, Sonia Shinhmar, Qingyu Zeng, Olivier Pardo, Philip Beesley, and Nelofer Syed & Robin SB Williams*

**Supplementary methods**

| **Sesn fw** | AATAATGAGAGATCCAGGAC |
| --- | --- |
| **Sesn Rev** | GCAGTATTTTCGGTTGCAG |
| **Oligo 1 fw** | 5’AGCAACGAGGTGATGCACGTTGGTTACA |
| **Oligo 1 rev** | 5’AAACAAACTGTAACCAACGTGCATCACC |
| **Oligo 2 fw** | 5’AGCA ACGATGCTTGGTCCATCGCTGAAT |
| **Oligo 2 rev** | 5’AAAC AAACATTCAGCGATGGACCAAGCA |
| **Oligo 3 fw** | 5’AGCA AGCAGGCATGAACCAATTCAGCGA |
| **Oligo 3 rev** | 5’AAAC AAACTCGCTGAATTGGTTCATGCC |

**Supplementary methods 1.1:** CRISPR knockout and sequencing primers.

| Primer | Fw (5’-3’) | Rev (5’-3’) |
| --- | --- | --- |
| Human Sestrin2 | GTGACCCCCTCAGCTGACAT | GAGCCCCTCTCCGAGTGAAG |
| Human PTEN | GTGGCGGAACTTGCAATCCT | CCGTCGTGTGGGTCCTGAAT |
| Human HPRT | CTCCGTTATGGCGACCC | CACCCTTTCCAAATCCTCAG |
| Mouse Sestrin2 | CCGCCACTCAGAGAAGGTTCA | CTGCGTACGGGGTAGTCAGG |
| Mouse PTEN | TCCTGCAGAAAGACTTGAAGGTG | CAAAAGGATACTGTGCAACTCTGC |
| Mouse HPRT | GCCATCACATTGTGGCCCTC | ATGTAATCCAGCAGGTCAGCAAAG |
| Dictyostelium Sesn | TTGGCTCATCAACCTTGGCTATTG | TTCAGCGATGGACCAAGCATCG |
| Dictyostelium mcfQ | CACCGTAGAAGCTTTAGGACATGC | AAGGCAATAGCGGCCATACCTG |

**Supplementary methods 1.2:** qPCR primers.

**Supplementary figures**


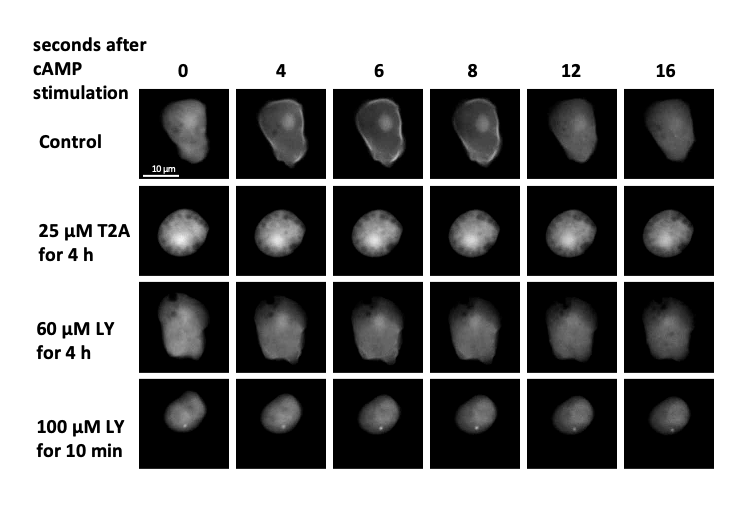


**Supplementary Figure S1: T2A and the PI3K inhibitor LY294002 (4h) suppress the activity of PI3K.** *D. discoideum* wild type cells were repeatedly stimulated with cAMP (6 min pulses of 100 nM) to force cells into an early development stage. Cells were either treated for 4 h with 25 µM T2A or with 60 µM PI3K inhibitor (LY294002) during the stimulation process or with 100 µM PI3K inhibitor for 10 min prior to the experiment. To determine the activity of PI3K cells were stimulated with 10 µM cAMP and the temporal membrane translocation of PH_Crac_-GFP was assessed by time‑lapse imaging.


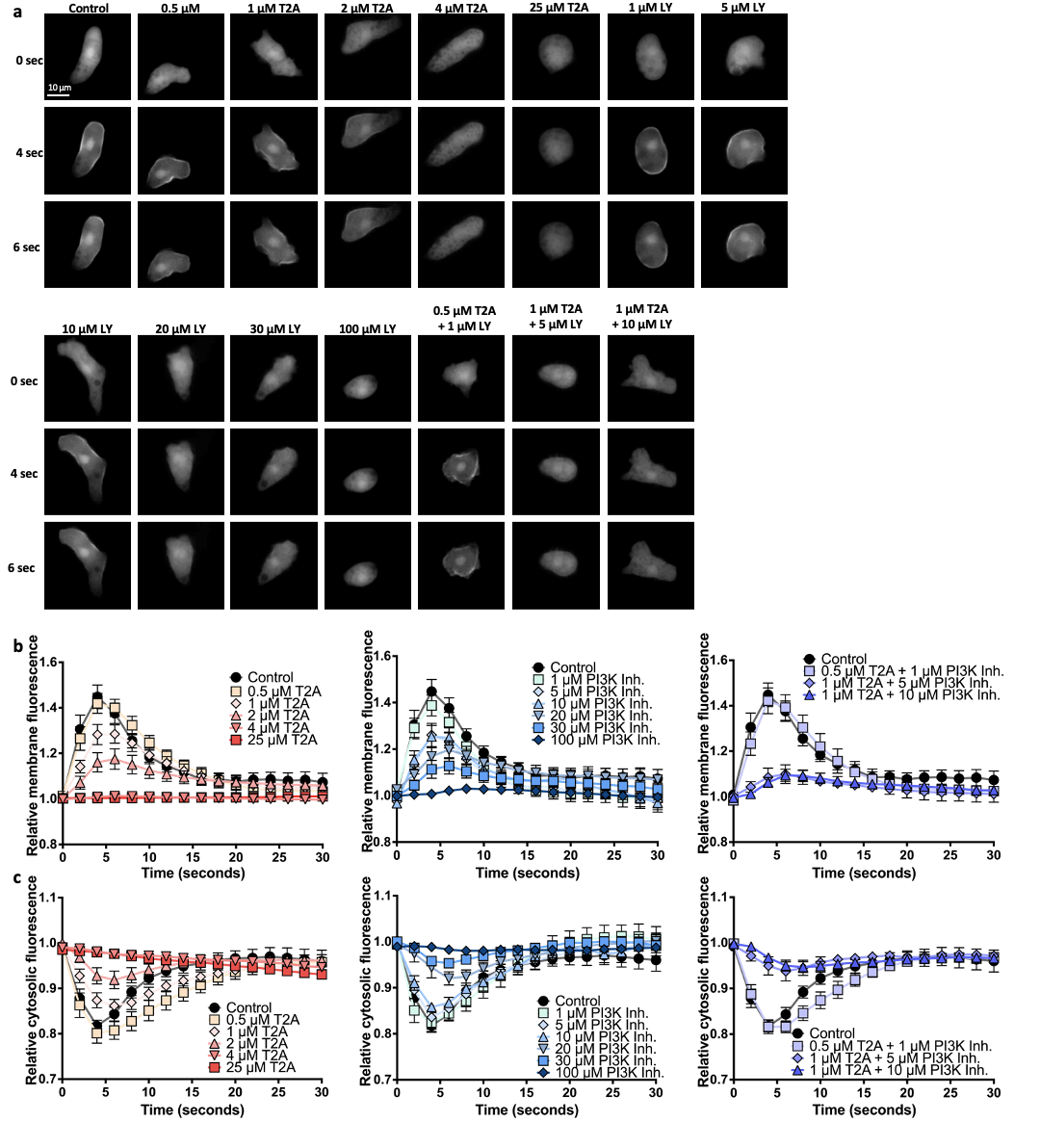


**Supplementary Figure S2: Dose-dependent acute inhibitory effects of T2A and the PI3K inhibitory on PI3K activity.** (A, B) *D. discoideum* wild type cells, which were forced into an early development stage by repeated stimulation with cAMP (6 min pulses of 100 nM for 4 h) were subsequently treated for 15 min with increasing concentrations of T–A (0 ‑ 25 µM) and of a PI3K inhibitor (LY2940–2, 0 ‑ 100 µM). After cells were stimulated with 10 µM cAMP the activity of PI3K was determined by assessing the temporal membrane translocation of PH_Crac_-GFP via time‑lapse imaging. (C) For statistical analysis the lowest (minimum) cytosolic fluorescence values were determined for each cell. All data are shown as mean ± SEM; * p ≤ 0.05, ** p ≤ 0.01, *** p ≤ 0.001, ns (not significant, p > 0.05) (two‑tailed Mann‑Whitney test).

| Combination (µM) | Y_ab_,O | Y_ab_,P | Synergy |
| --- | --- | --- | --- |
| T2A 0.5  LY 1.0 | 0.01 | 0.12 | No |
| T2A 1.0  LY 5.0 | 0.24 | 0.1 | Yes |
| T2A 1.0  LY 10.0 | 0.25 | 0.21 | Yes |

**Supplementary Figure S3: BLISS analysis of PI3K inhibition from PIP3 assay shows synergy between T2A and LY294002.** Combinations of T2A and LY294002 have greater observed inhibition of PI3K than predicted inhibition, suggesting synergy.


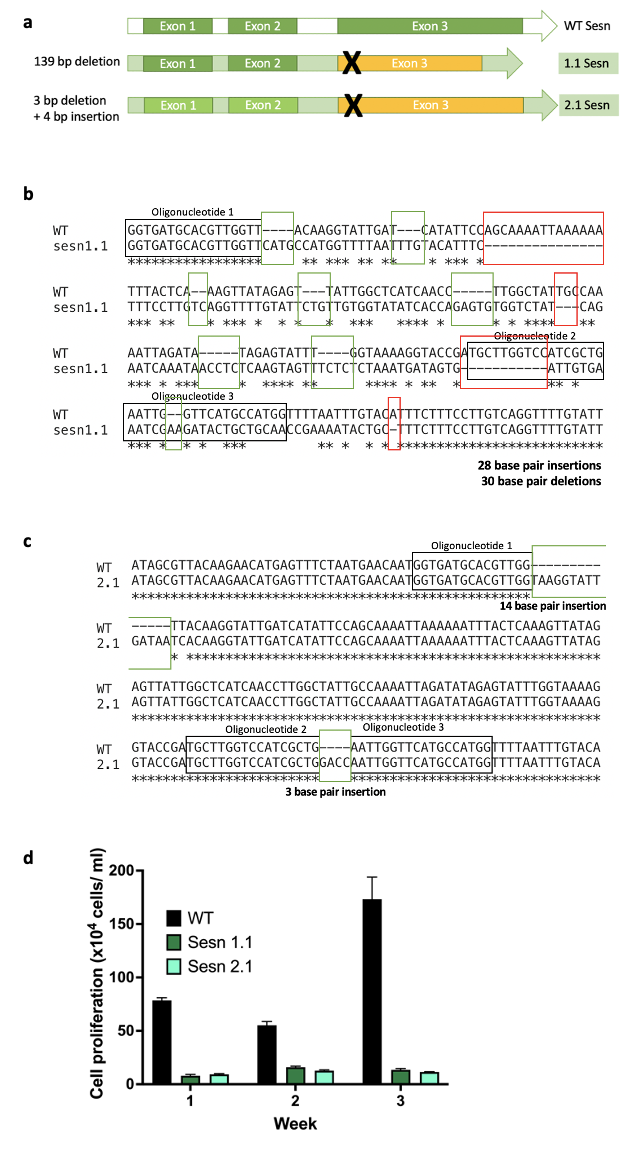


**Supplementary Figure S4: Ablation of sesn gene in independent cells reduces cell proliferation.** Mutant solvent control cells grew slower than wild type with an average maximum growth of the wild type cells reaching a 95 x10^4^ cell /ml whilst the sesn mutant had maximum 18 x10^4^ cells/ml.

**
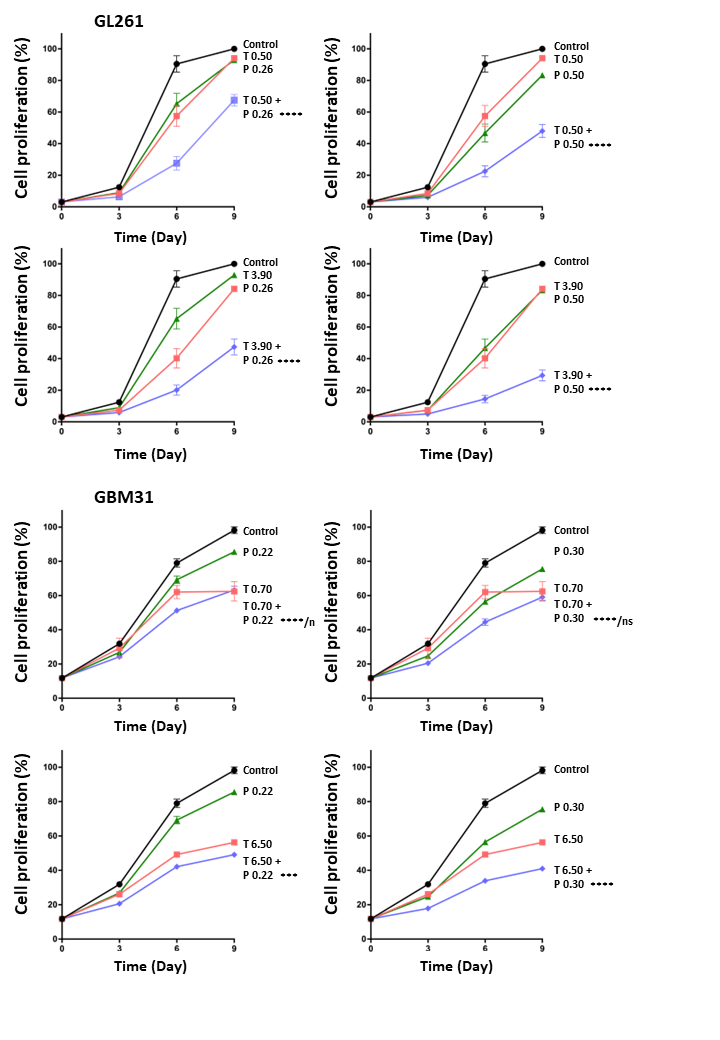
**

**Supplementary Figure S5** …. To be completed 2 D cell proliferation assays time dependence


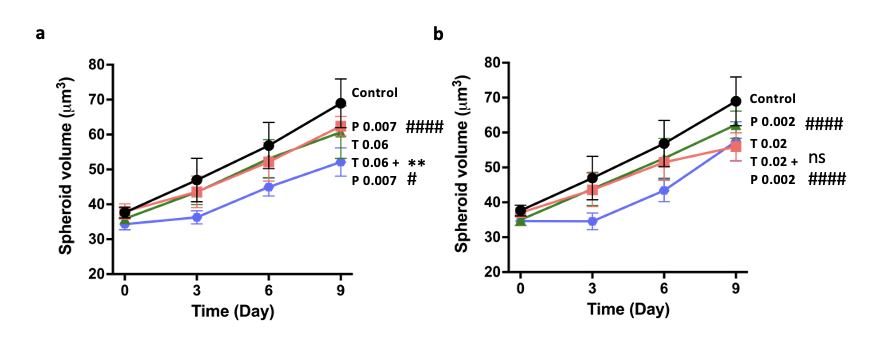


**Supplementary Figure S6: T2A enhances Paxalisib-dependent inhibition of human-derived GBM spheroid expansion.** (A) GBM59, primary human glioblastoma cells were cultured into 3D spheroids which were treated with (A) solvent only control, T2A (0.37 µM) and Paxalisib (0.05 µM) or (B) T2A (0.02 µM) and Paxalisib (0.003 µM) where each compound treatment individually decreased spheroid growth compared to solvent control. All data are shown as mean ± SEM; * p ≤ 0.05, ** p ≤ 0.01, *** p ≤ 0.001, ns (not significant, p > 0.05) (two‑tailed Mann‑Whitney test).


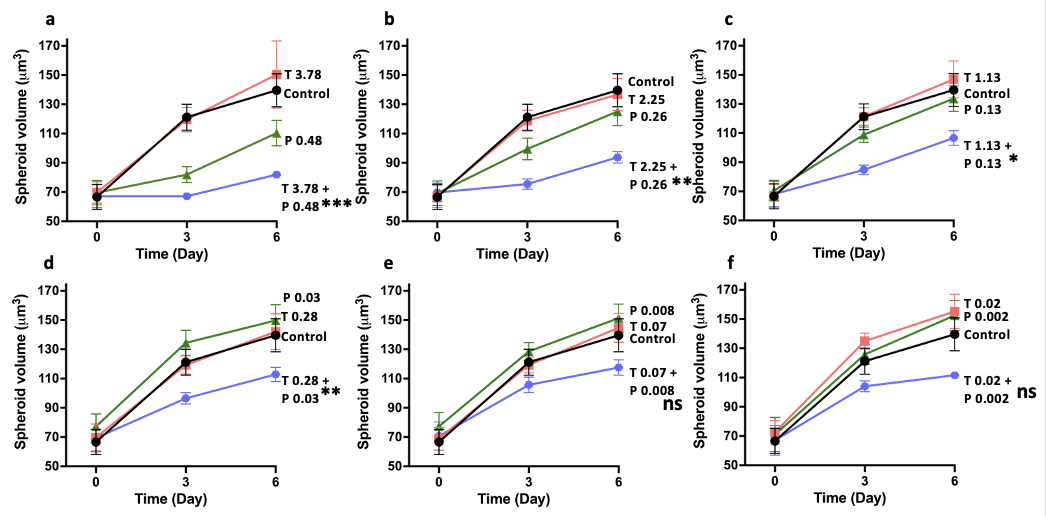


**Supplementary Figure S7: T2A in combination with PI3K inhibitor (Paxalisib/GDC-0085) inhibit the growth of 3D mouse derived GMB spheroids*.*** GL261 (mouse) spheroid volume was measured and calculated at days 0, 3 and 6. (A-F) Spheroids were exposed to a range of T2A (0.02-3.78 µM) and Paxalisib (0.002- 0.48µM) and combinatory treatments where combinations were always more effective in decreasing spheroid growth than single compound treatment alone. All data are shown as mean ± SEM; * p ≤ 0.05, ** p ≤ 0.01, *** p ≤ 0.001, ns (not significant, p > 0.05) (two‑tailed Mann‑Whitney test).

| Cell line | Combination (µM) | Y_ab_,O | Y_ab_,P | Synergy |
| --- | --- | --- | --- | --- |
| GBM59 | T 3.77  P 0.46 | 1.36 | 0.97 | Yes |
|  | T 1.84  P 0.22 | 1.22 | 0.96 | Yes |
|  | T 0.92  P 0.11 | 0.95 | 0.90 | Yes |
|  | T 0.23  P 0.03 | 0.79 | 0.66 | Yes |
|  | T 0.06  P 0.007 | 0.44 | 0.38 | Yes |
|  | T 0.014  P 0.002 | 0.28 | 0.47 | No |
| GL261 | T 3.78  P 0.48 | 0.80 | 0.52 | Yes |
|  | T 2.25  P 0.26 | 0.68 | 0.27 | Yes |
|  | T 1.13  P 0.13 | 0.48 | 0.06 | Yes |
|  | T 0.28  P 0.03 | 0.41 | 0.02 | Yes |
|  | T 0.07  P 0.008 | 0.36 | -0.04 | Yes |
|  | T 0.02  P 0.002 | 0.40 | -0.25 | Yes |

**Supplementary Figure S8: BLISS value calculations show synergistic effect of tanshinone IIA and Paxalisib inhibition of spheroid growth.** GBM59 and GL261 cells in 3D cell culture were exposed to tanshinone IIA, Paxalisib or a combination treatment. The BLISS values were calculated comparing the observed inhibition of combination treatment to predicted inhibition where observed inhibition was greater than predicted, proving synergy. All data are shown as mean ± SEM; * p ≤ 0.05, ** p ≤ 0.01, *** p ≤ 0.001, ns (not significant, p > 0.05) (two‑tailed Mann‑Whitney test).
