# Supplementary material for: Enhanced Sestrin expression through Tanshinone 2A treatment improves PI3K-dependent inhibition of glioma growth
Source: Cell Death Discov. 2023 May 19;9:172. doi: 10.1038/s41420-023-01462-6 (PMC10195868; doi:10.1038/s41420-023-01462-6)

Westerns

p-PKB WT 1 hr

Cell type: WT and PKB $^{-/-}$ *D. discoideum*
Treatment: 1 hour, 25 µM Tanshinone IIA or DMSO solvent control
Antibody: phospho-PKB substrate 1:1000, CST 10001 23C8D2

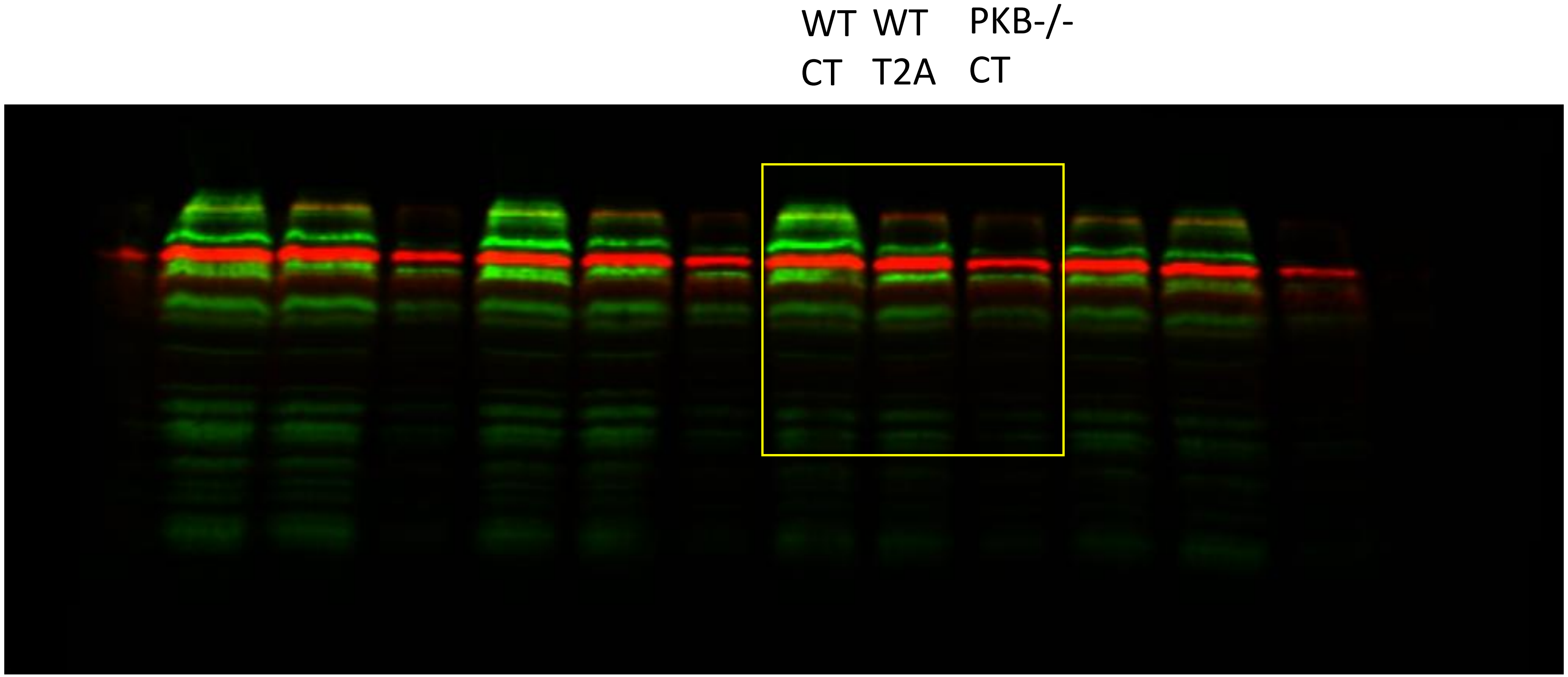

p-PKB WT 24 hr

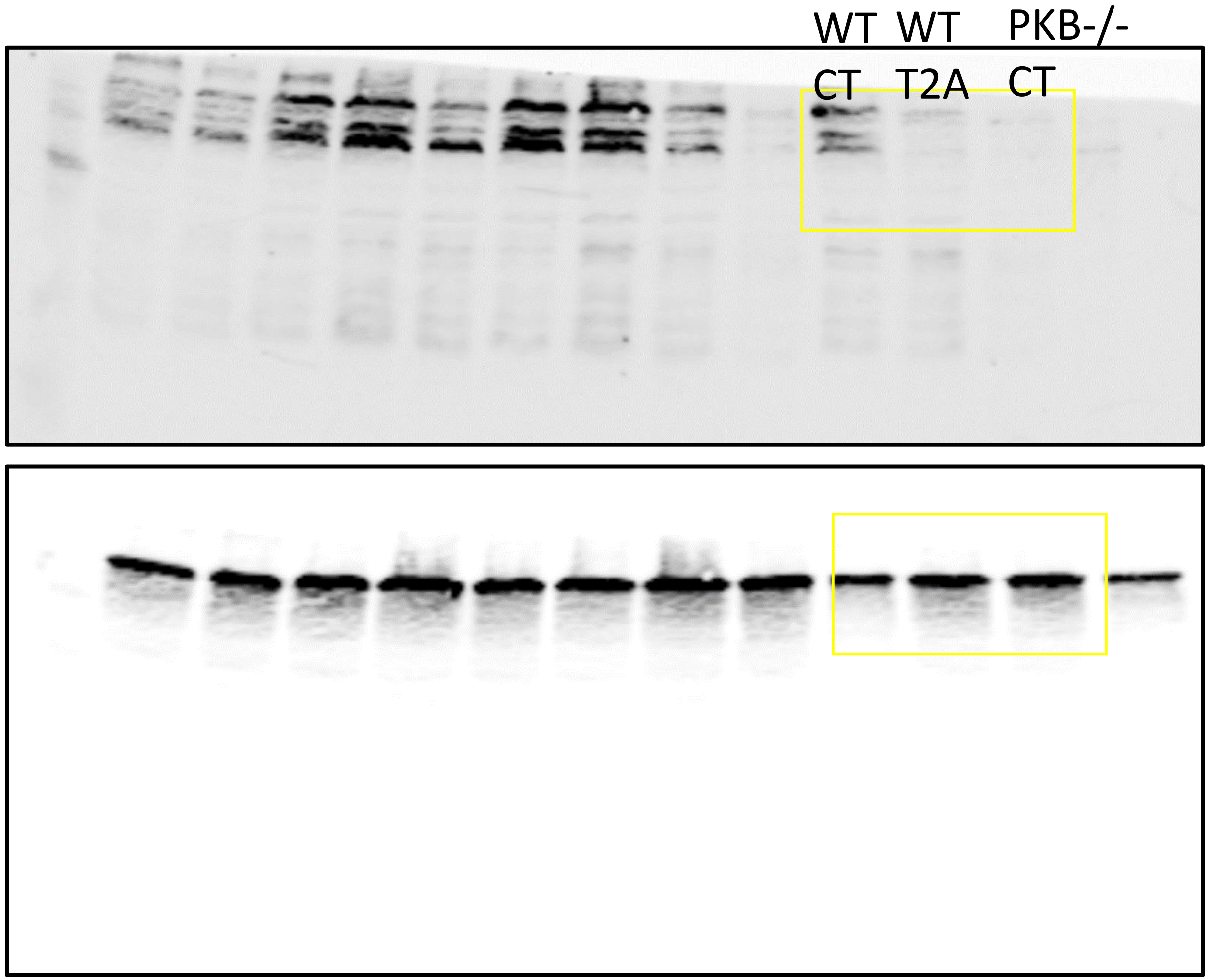

Cell type: WT and PKB $^{-/-}$ *D. discoideum*
Treatment: 24 hour, 25 µM Tanshinone IIA or DMSO solvent control
Antibody: phospho-PKB substrate 1:1000, CST 10001 23C8D2

p-4EBP1 WT 1 hr

Cell type: WT *D. discoideum*
Treatment: 1 hour, 25 µM Tanshinone IIA or DMSO solvent control
Antibody: p-(Thr37/46)4EBP1, 1:1000, CST 9459

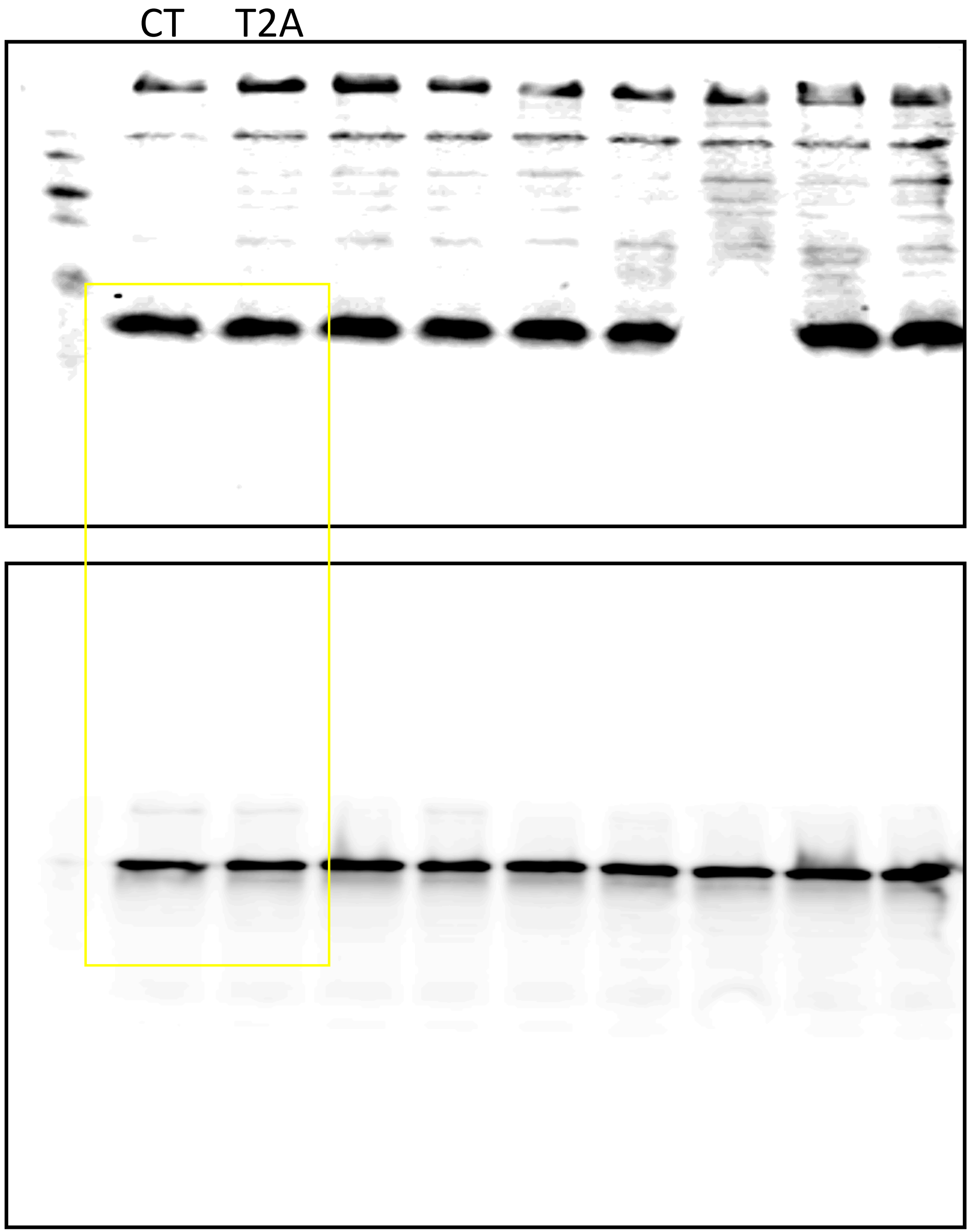

p-4EBP1 WT 24 hr

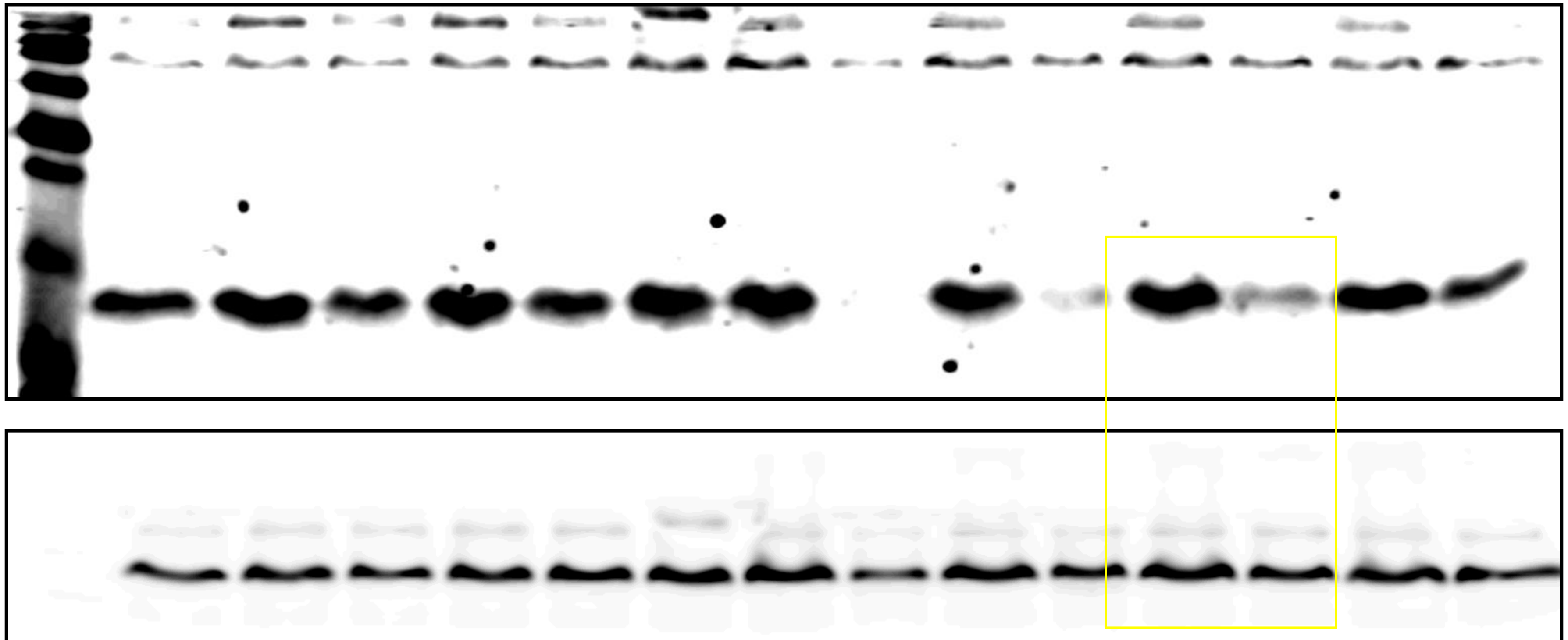

Cell type: WT *D. discoideum*
Treatment: 24 hour, 25 µM Tanshinone IIA or DMSO solvent control
Antibody: p-(Thr37/46)4EBP1, 1:1000, CST 9459

p-4EBP1 PKB-/- 24 hr

Cell type: PKB$^{-/-}$ *D. discoideum (mutant)*
Treatment: 24 hour, 25 µM Tanshinone IIA or DMSO solvent control
Antibody: p-(Thr37/46)4EBP1, 1:1000, CST 9459

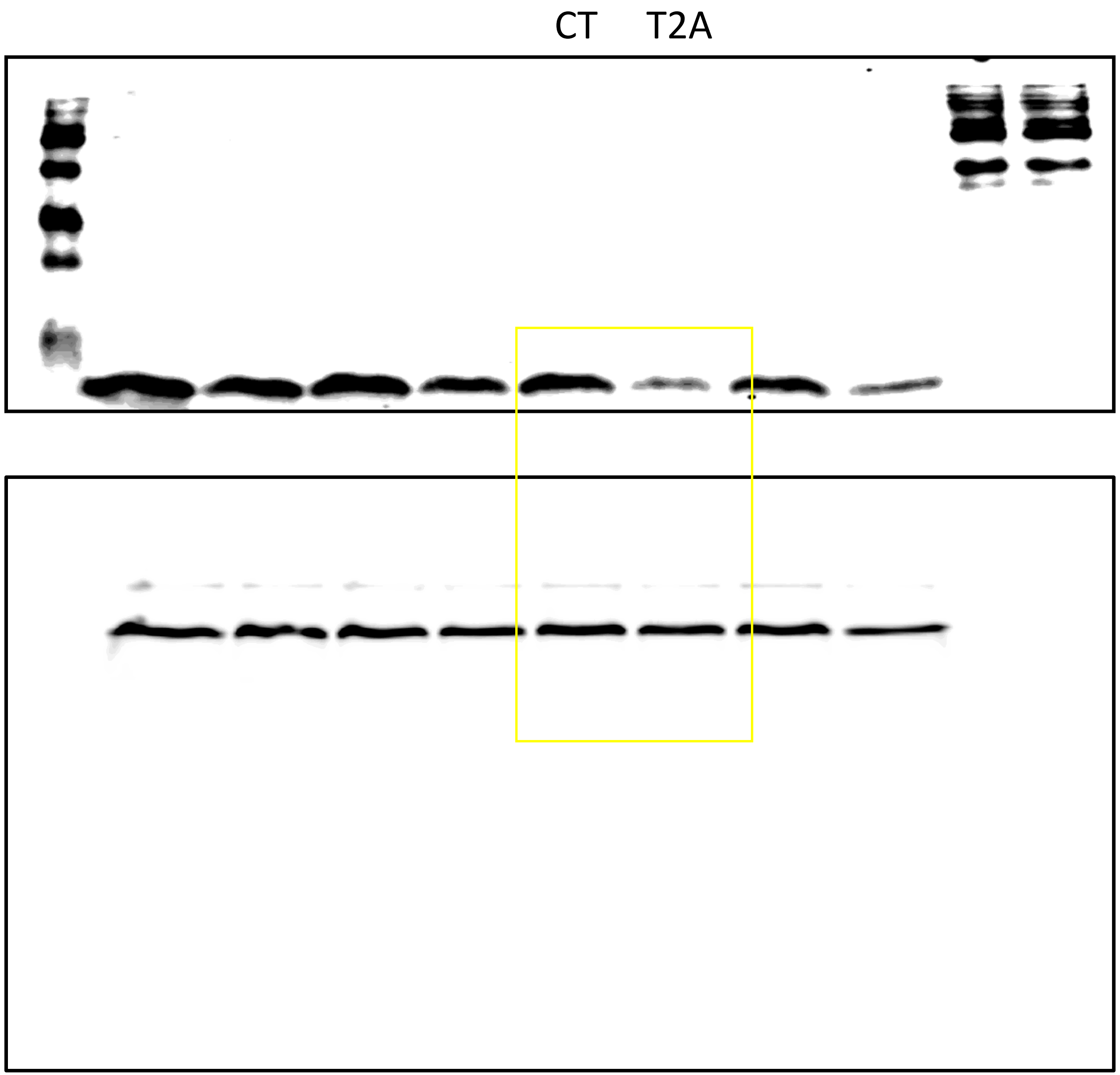

p-4EBP1 tsc2-

Cell type: tsc2$^-$ *D. discoideum (mutant)*
Treatment: 24 hour, 25 μM Tanshinone IIA or DMSO solvent control
Antibody: p-(Thr37/46)4EBP1, 1:1000, CST 9459

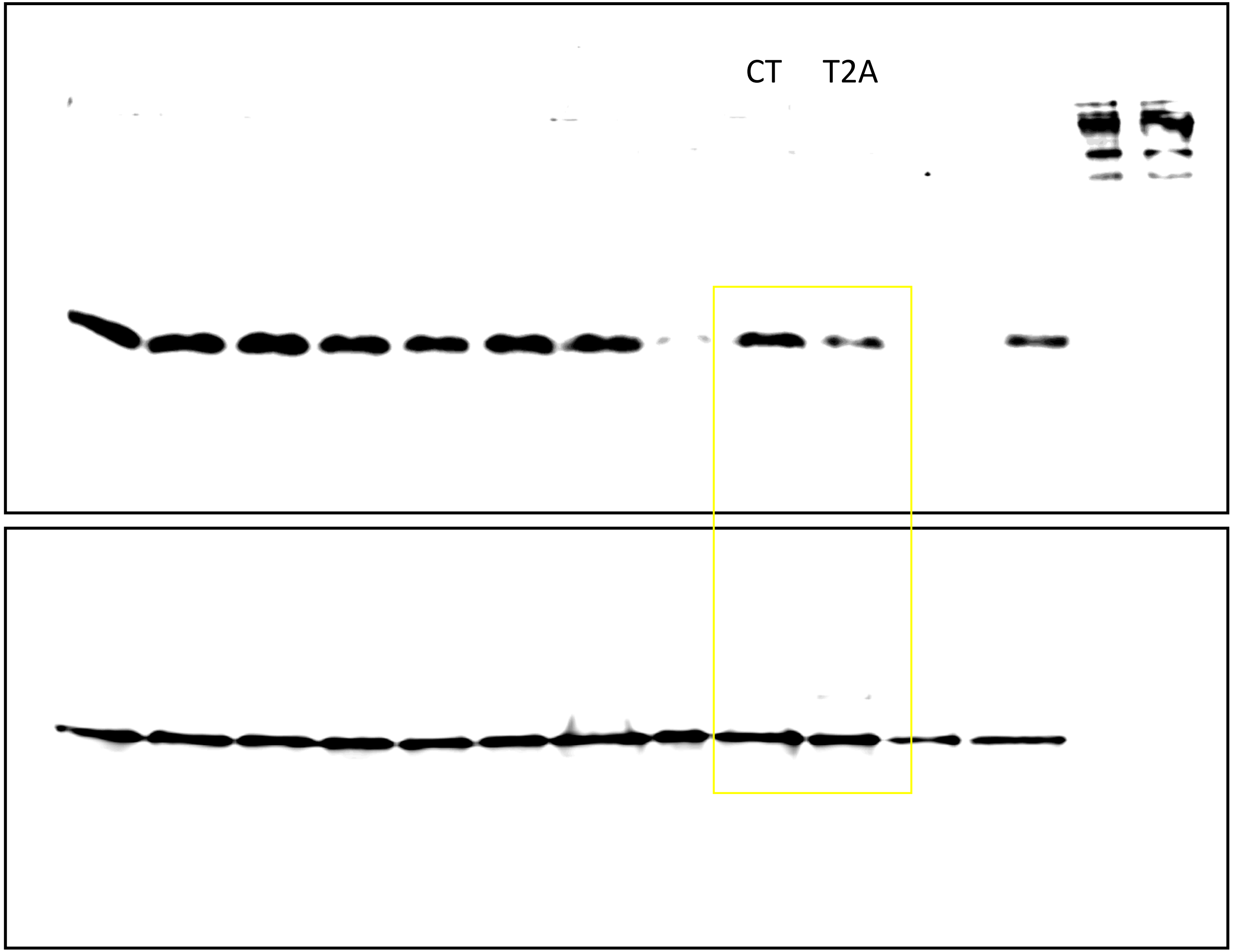

p-4EBP1 snfA- 24 hr

Cell type: snfA[-] *D. discoideum (mutant)*
Treatment: 24 hour, 25 µM Tanshinone IIA or DMSO solvent control
Antibody: p-(Thr37/46)4EBP1, 1:1000, CST 9459

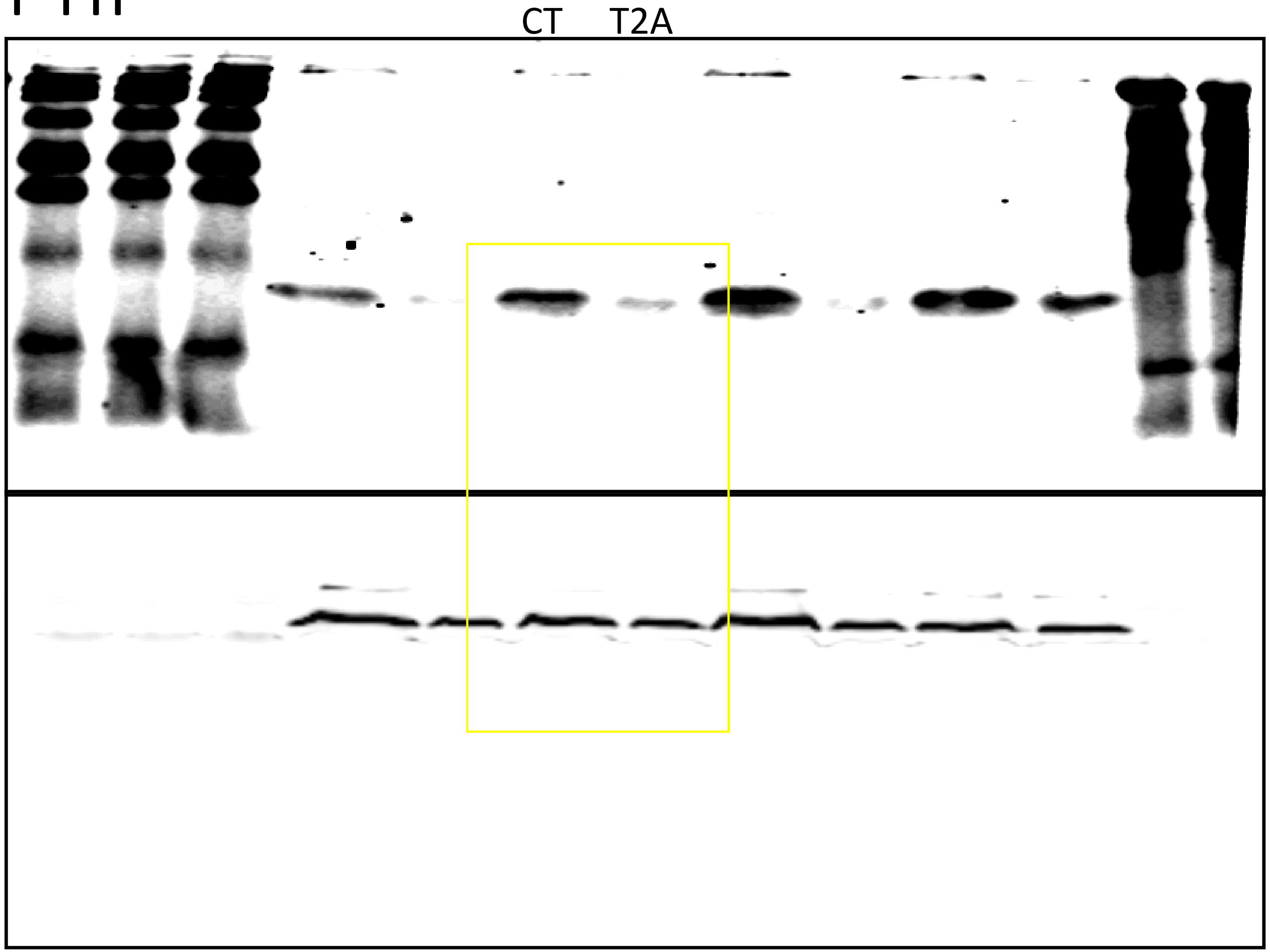

p-4EBP1 WT combination 1 hr

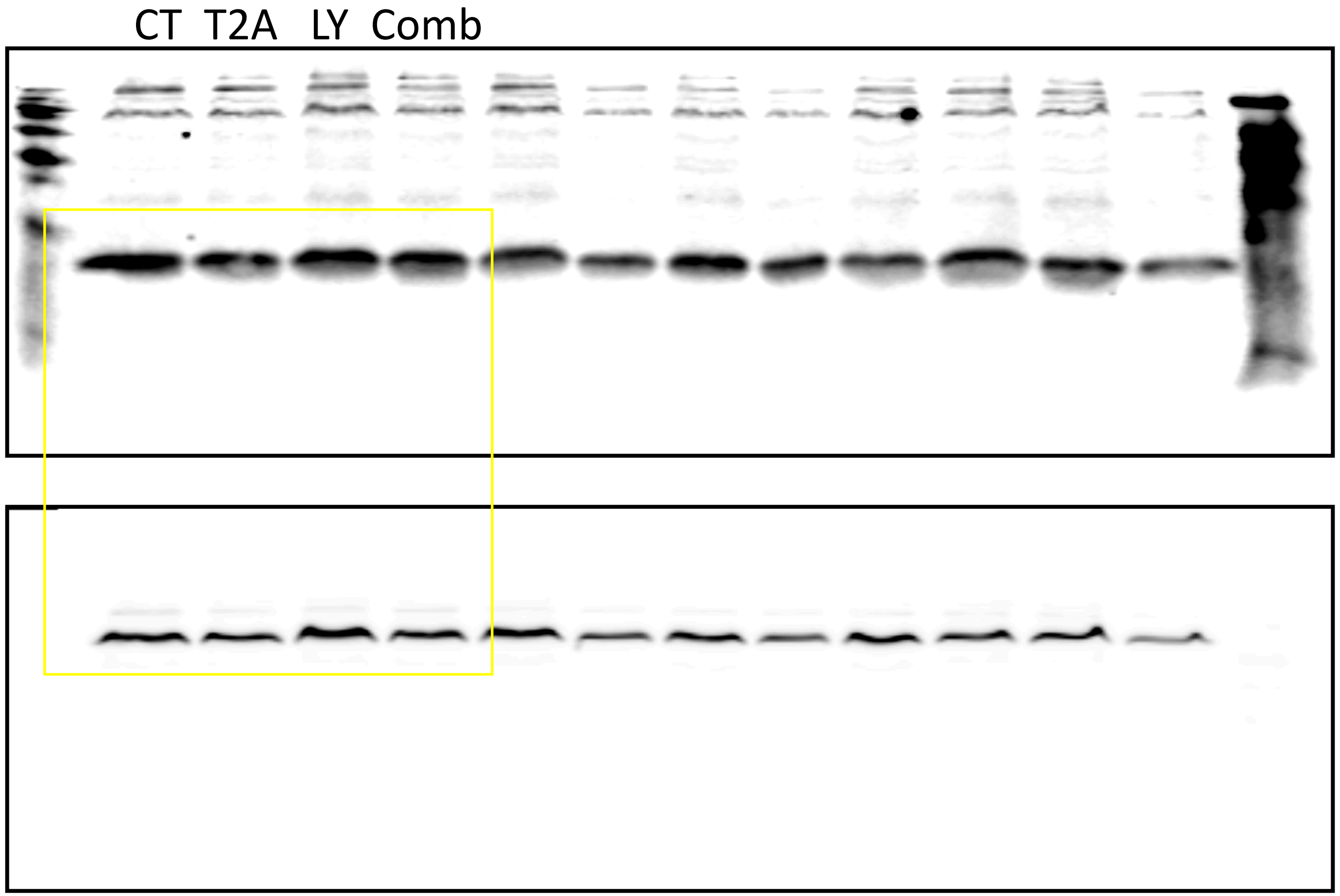

Cell type: WT *D. discoideum*
Treatment: 1 hour, DMSO solvent control, 12 µM Tanshinone IIA, 14 µM LY294002 or combination of 12 µM T2A + 14 µM LY294002
Antibody: p-(Thr37/46)4EBP1, 1:1000, CST 9459

p-4EBP1 WT combination 5 days

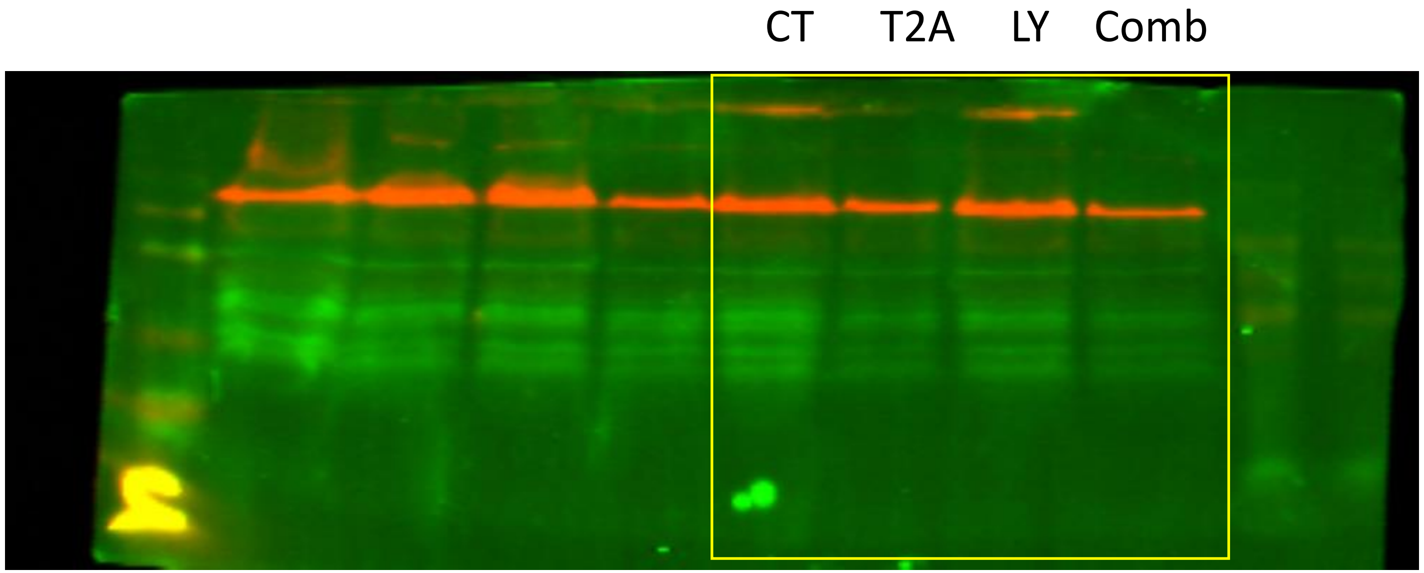

Cell type: WT *D. discoideum*
Treatment: 5 days, DMSO solvent control, 12 µM Tanshinone IIA, 14 µM LY294002 or combination of 12 µM T2A + 14 µM LY294002
Antibody: p-(Thr37/46)4EBP1, 1:1000, CST 9459

p-4EBP1 sestrin 24 hr

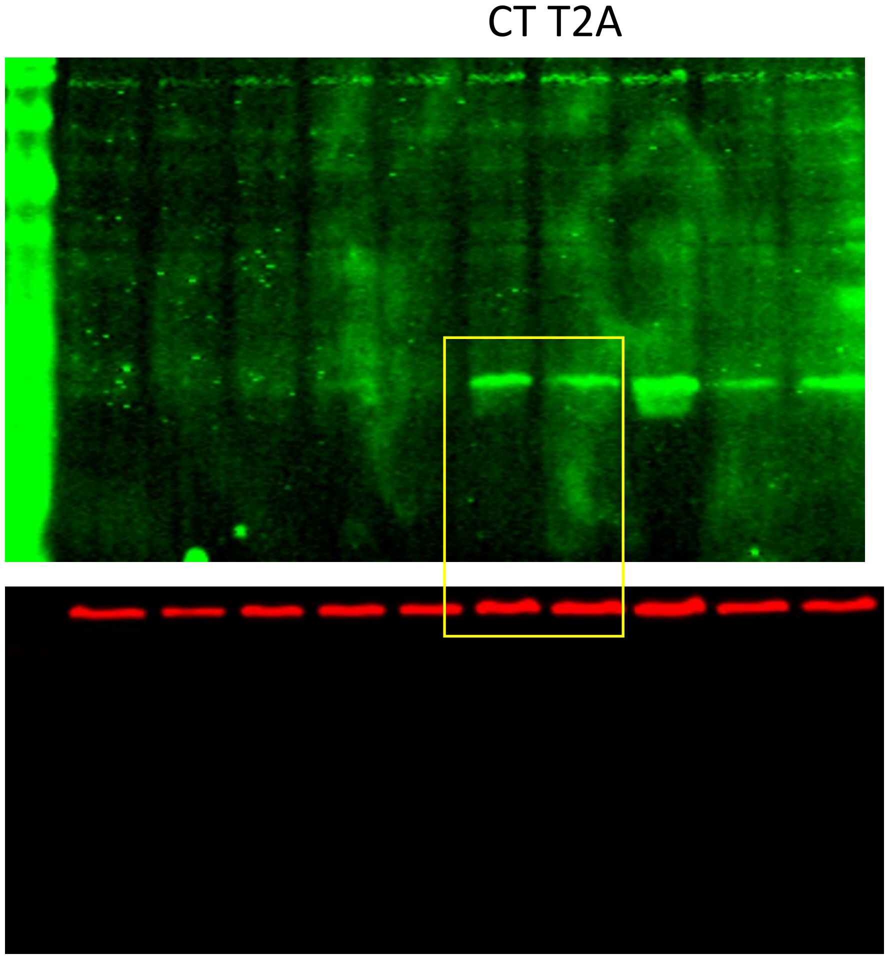

Cell type: sesn$^-$ *D. discoideum (mutant)*
Treatment: 24 hour, DMSO solvent control or 25 μM Tanshinone IIA
Antibody: p-(Thr37/46)4EBP1, 1:1000, CST 9459

Sestrin2 GBM59

Cell type: GBM59 (primary human GBM cell line)
Treatment: 3 days , DMSO solvent control,2 3.77 µM Tanshinone IIA, 0.46 µM Paxalisib or combination of 3.77 µM T2A + 0.46 µM Paxalisib
Antibody: sestrin2 polyclonal antibody, ProteinTech, 10795/ B-actin, Sigma, A228

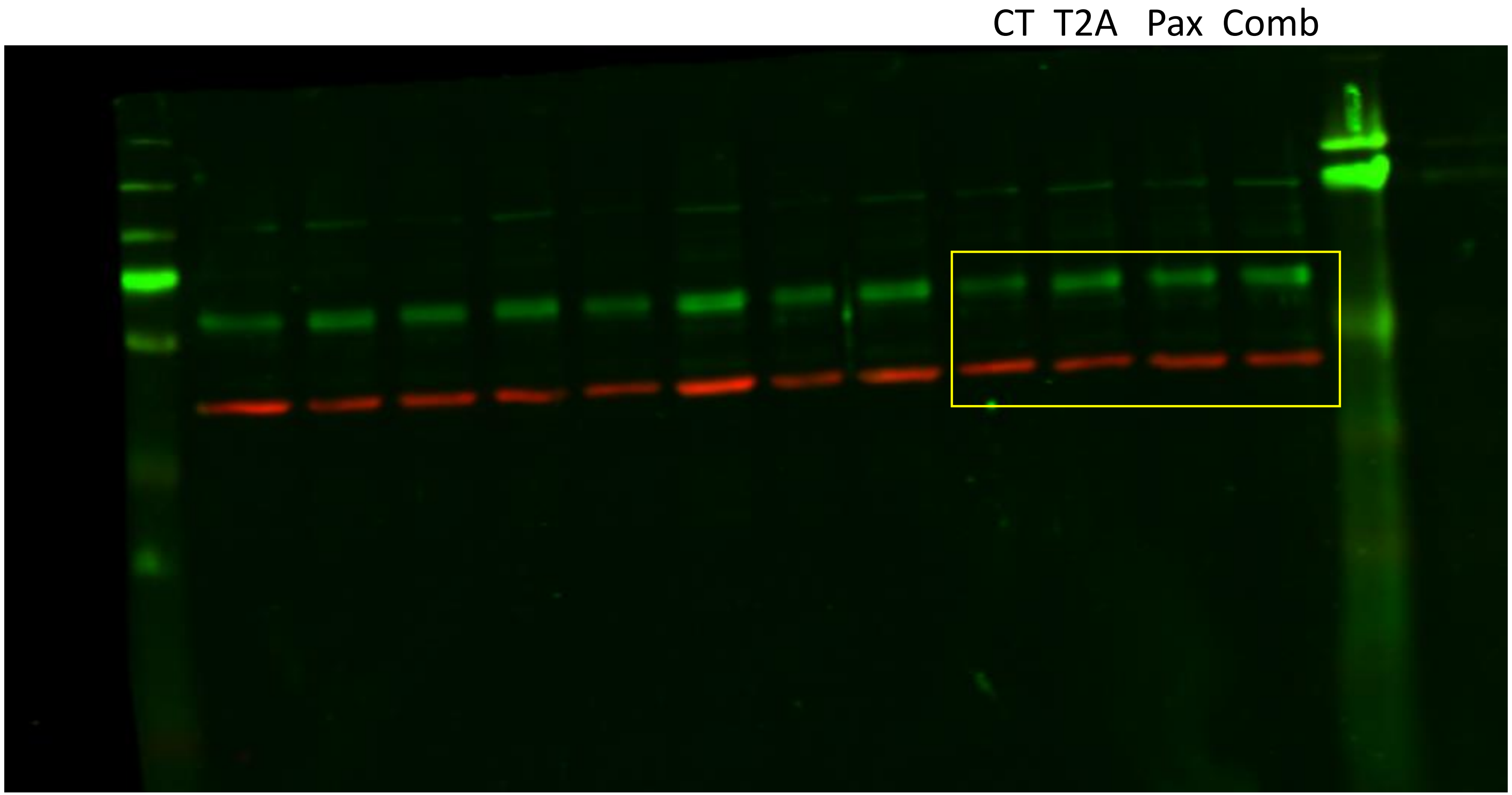

Sestrin2 GL261

Cell type: GL261 (mouse GBM cell line)
Treatment: 3 days , DMSO solvent control,2 3.78 µM Tanshinone IIA, 0.48 µM Paxalisib or combination of 3.78 µM T2A + 0.48 µM Paxalisib
Antibody: sestrin2 polyclonal antibody, ProteinTech, 10795/ B-actin, Sigma, A228

CT T2A Pax Comb

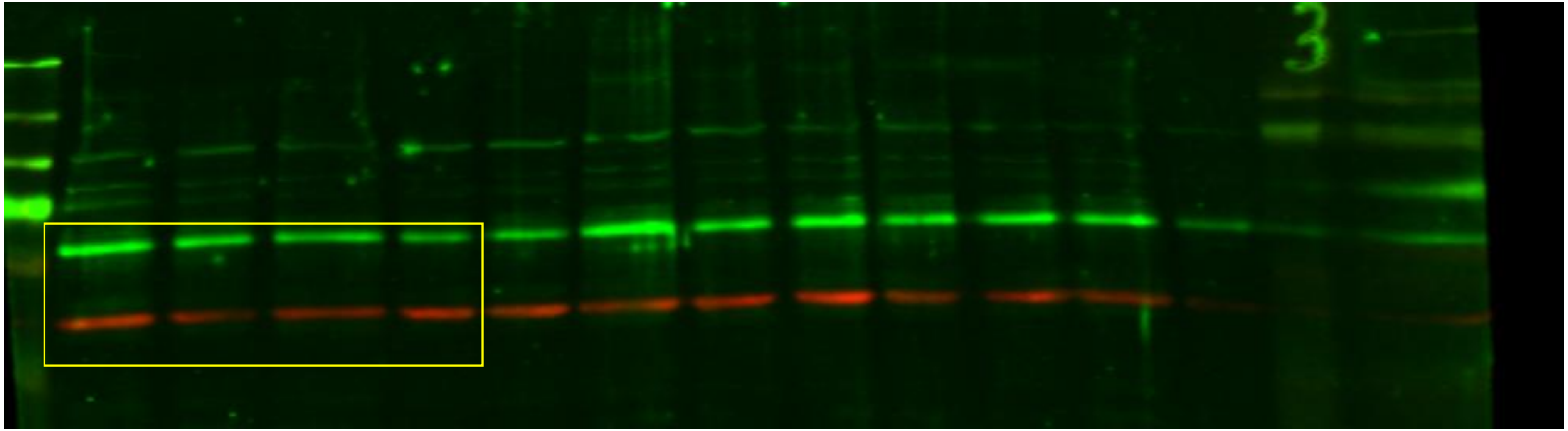

p53 GBM59

Cell type: GBM59 (primary human GBM cell line)
Treatment: 3 days , DMSO solvent control,2 3.77 µM Tanshinone IIA, 0.46 µM Paxalisib or combination of 3.77 µM T2A + 0.46 µM Paxalisib
Antibody: p53 antibody, CST, 9282T/ B-actin, Sigma, A228

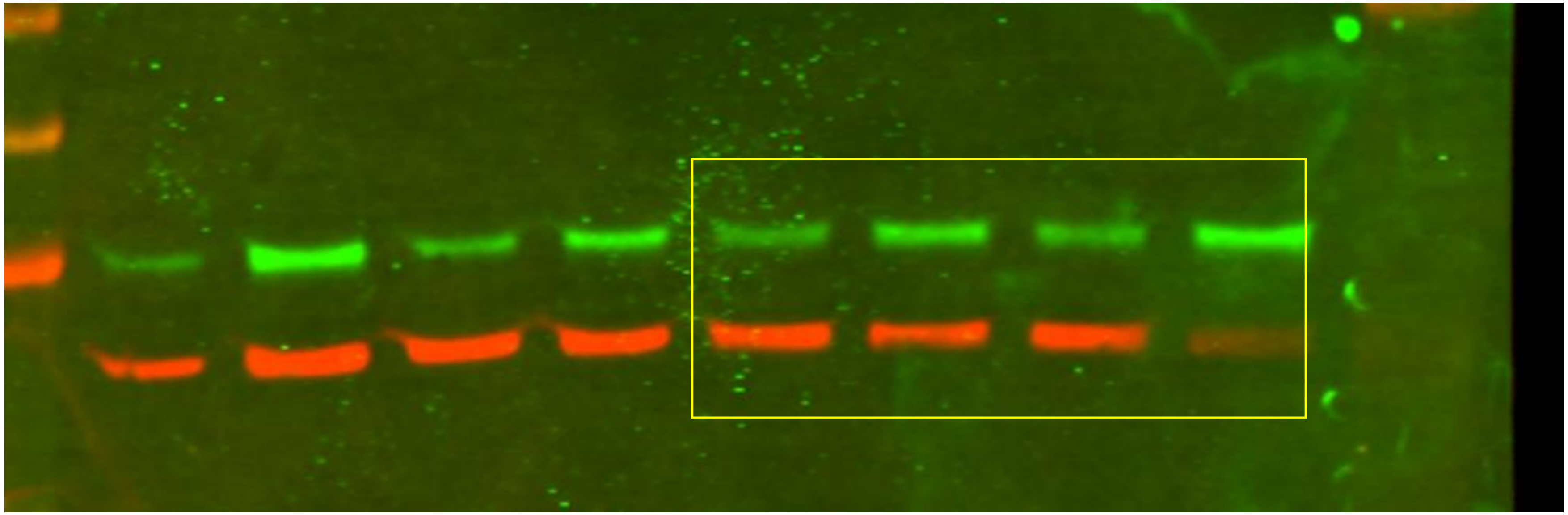

p53 GL261

Cell type: GL261 (mouse GBM cell line)
Treatment: 3 days , DMSO solvent control,2 3.78 µM Tanshinone IIA, 0.48 µM Paxalisib or combination of 3.78 µM T2A + 0.48 µM Paxalisib
Antibody: p53 antibody, CST, 9282T/ B-actin, Sigma, A228

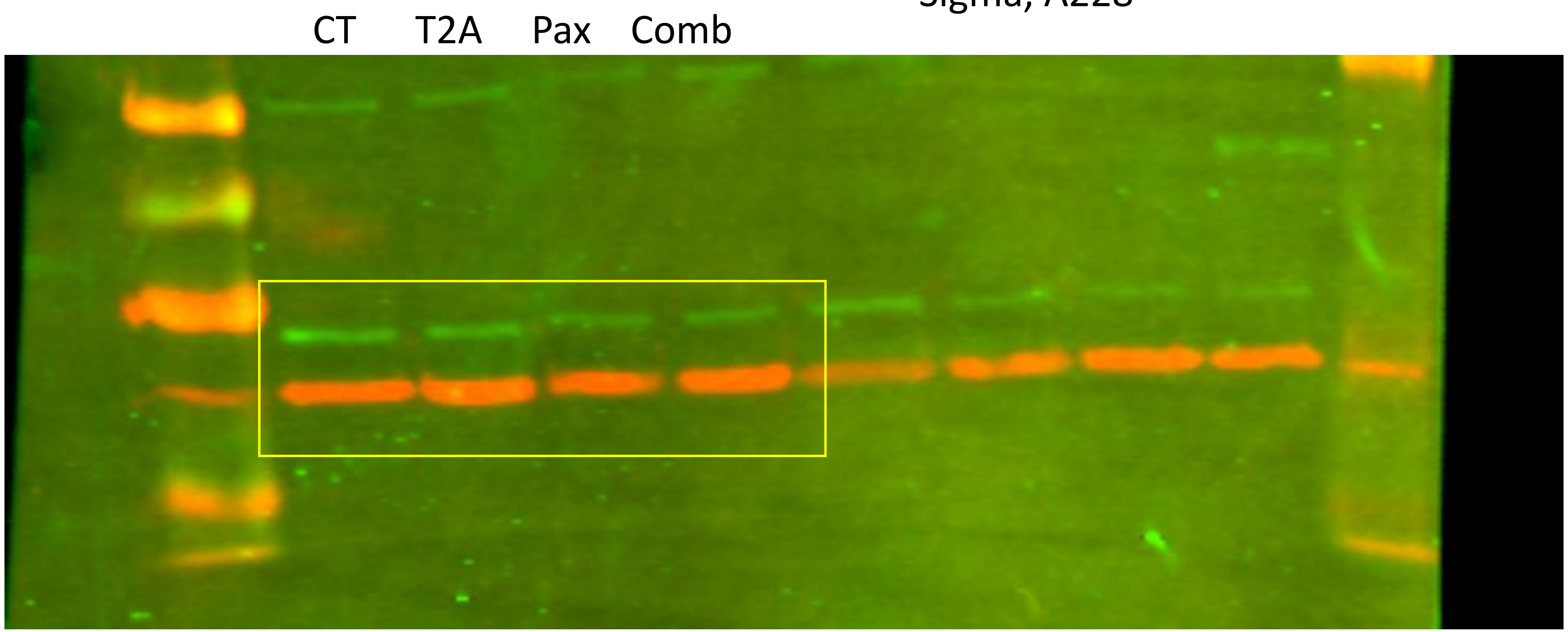

GefS 1 hr

Cell type: WT and PKB $^{-/-}$ *D. discoideum*
Treatment: 1 hr, DMSO solvent control, 25 µM Tanshinone IIA
Antibody: p-GefS substrate, CST, 110B7E

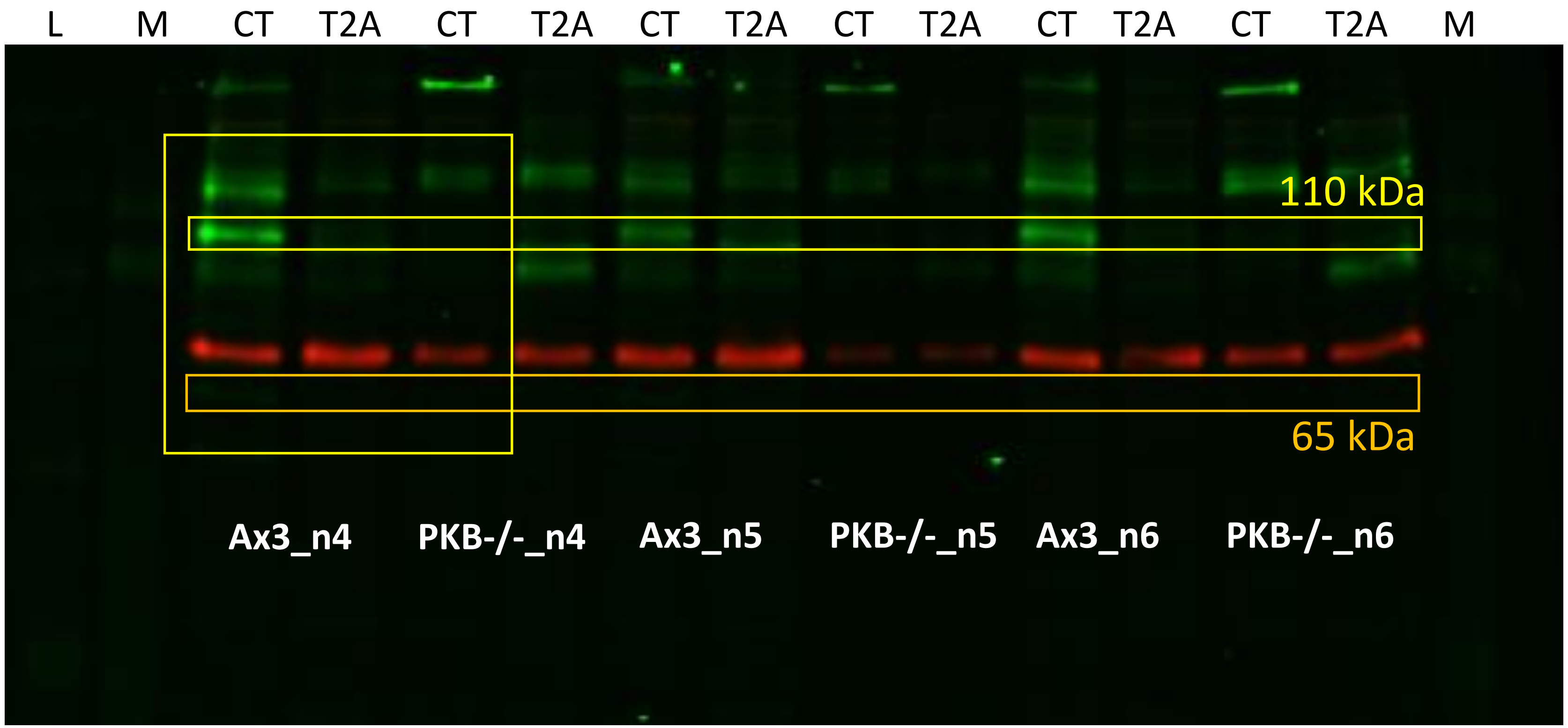

GefS WT 24 hr

Cell type: WT and PKB $^{-/-}$ *D. discoideum*
Treatment: 24 hr, DMSO solvent control, 25 µM Tanshinone IIA
Antibody: p-GefS substrate, CST, 110B7E

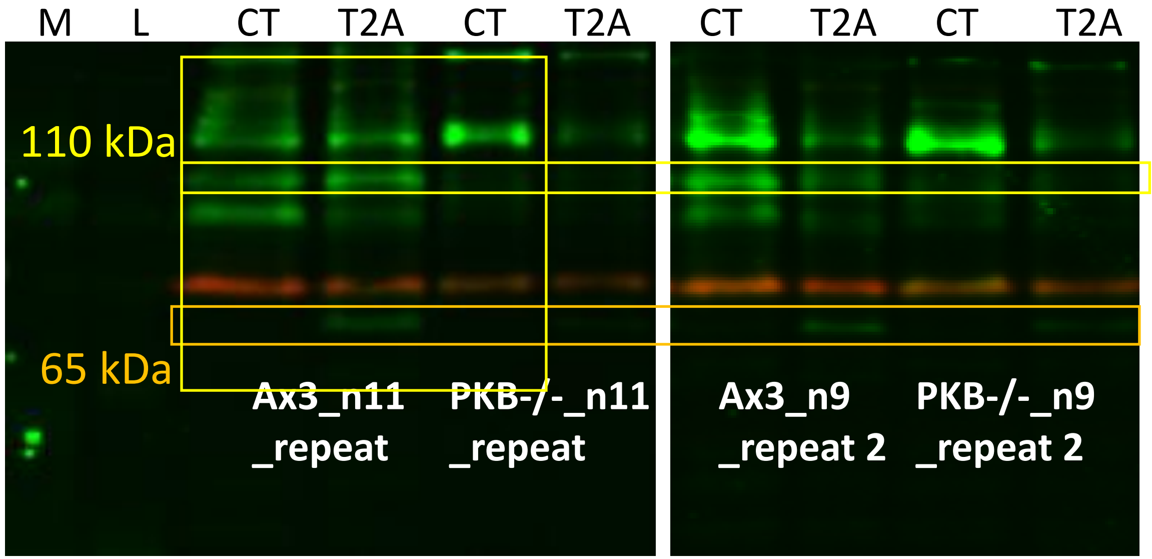

Supplement: Supplementary file 2 — Original Data File [file 41420_2023_1462_MOESM2_ESM.pdf]
